# Aromatase inhibition 2013: clinical state of the art and questions that remain to be solved

**DOI:** 10.1530/ERC-13-0099

**Published:** 2013-08

**Authors:** Per Eystein Lønning, Hans Petter Eikesdal

**Affiliations:** 1 Section of Oncology, Department of Clinical ScienceUniversity of BergenBergenNorway; 2 Department of OncologyHaukeland University HospitalJonas Lies vei 26N-5021, BergenNorway

**Keywords:** breast cancer, endocrine therapy, aromatase inhibitors, adjuvant therapy, resistance

## Abstract

Following their successful implementation for the treatment of metastatic breast cancer, the ‘third-generation’ aromatase inhibitors (anastrozole, letrozole, and exemestane) have now become standard adjuvant endocrine treatment for postmenopausal estrogen receptor-positive breast cancers. These drugs are characterized by potent aromatase inhibition, causing >98% inhibition of estrogen synthesis *in vivo*. A recent meta-analysis found no difference in anti-tumor efficacy between these three compounds. As of today, aromatase inhibitor monotherapy and sequential treatment using tamoxifen followed by an aromatase inhibitor for a total of 5 years are considered equipotent treatment options. However, current trials are addressing the potential benefit of extending treatment duration beyond 5 years. Regarding side effects, aromatase inhibitors are not found associated with enhanced risk of cardiovascular disease, and enhanced bone loss is prevented by adding bisphosphonates in concert for those at danger of developing osteoporosis. However, arthralgia and carpal tunnel syndrome preclude drug administration among a few patients. While recent findings have questioned the use of aromatase inhibitors among overweight and, in particular, obese patients, this problem seems to focus on premenopausal patients treated with an aromatase inhibitor and an LH-RH analog in concert, questioning the efficacy of LH-RH analogs rather than aromatase inhibitors among overweight patients. Finally, recent findings revealing a benefit from adding the mTOR inhibitor everolimus to endocrine treatment indicate targeted therapy against defined growth factor pathways to be a way forward, by reversing acquired resistance to endocrine therapy.

## Introduction

The history of endocrine therapy for advanced breast cancer started more than a decade ago with the seminal discovery by George Beatson (1896) that ovarian ablation may cause tumor regression in premenopausal women. While ovarian estrogen synthesis ceases at the menopause, postmenopausal women still have plasma estrogen levels present at low concentration. Previously believed to occur by adrenal glandular synthesis, it later became clear that the adrenals are contributors of circulating androgens, subsequently converted into estrogens in different body compartments (Lønning *et al*. 1990). The idea that estrogen ablation might also work in postmenopausal women triggered implementation of adrenalectomy as well as hypophysectomy in the 1950s (Luft *et al*. 1952, Huggins & Dao 1953, Fracchia *et al*. 1967, 1971).

The fact that adrenalectomy as well as hypophysectomy was an effective antitumor therapy albeit at a cost of high morbidity motivated trials on ‘medical adrenalectomy’, leading to testing of glucocorticoids (Kofman *et al*. 1958, Lemon 1959), as well as adrenal enzyme inhibitors like ketoconazole (Harris *et al*. 1988). While the response to these drugs was inferior to surgical adrenalectomy/hypophysectomy, these attempts, by chance, paved the way for aminoglutethimide, subsequently leading to implementation of aromatase inhibition for breast cancer therapy.

The details of how aminoglutethimide was introduced for breast cancer therapy has been outlined elsewhere (Lønning & Kvinnsland 1988). It was originally developed as an unsuccessful antiepileptic compound causing significant adrenocortical toxicity. Following an initial clinical observation revealing efficacy of aminoglutethimide in a breast cancer patient by Ralph Cash *et al*. (1967), Richard Santen *et al*. (1974) systematically implemented aminoglutethimide in concert with glucocorticoid substitution in an attempt to achieve an effective medical adrenalectomy. Their studies demonstrated clinical efficacy of aminoglutethimide for the treatment of postmenopausal breast cancer (Santen *et al*. 1981). In addition, their systematic endocrine studies revealed surprising findings with respect to its mechanism of action. Contrary to expectations, they found adrenal androgen synthesis to be preserved (Samojlik *et al*. 1980) despite profound suppression of plasma estrogen levels (Santen 1981). Thus, in a seminal study, they confirmed aminoglutethimide to act as an aromatase inhibitor *in vivo* (Santen *et al*. 1978), introducing aromatase inhibition as a novel concept in breast cancer therapy. Studies conducted several years later revealed aminoglutethimide, in addition, to enhance estrogen metabolism (Lønning *et al*. 1987, 1989*a*).

In parallel, Harry and Angela Brodie experimentally worked on androstenedione derivatives for aromatase inhibition (Brodie *et al*. 1977, 1983), leading to the first pilot trial revealing anti-tumor efficacy of 4-hydroxyandrostenedione in metastatic breast cancer (Coombes *et al*. 1984).

Following development of aminoglutethimide and 4-hydroxyandrostenedione (known as lentaron), other so-called second-generation aromatase inhibitors, including fadrozole, were developed; for a detailed review of clinical studies evaluating first- and second-generation aromatase inhibitors for metastatic breast cancer, the readers are referred to a previous review (Lønning 2004). None of these compounds are in clinical use any longer. In brief, while some of these compounds, like fadrozole and 4-hydroxyandrostenedione, were associated with less side effects compared with standard treatment regimens at that time, anti-tumor effects were not superior to the effect of aminoglutethimide or other contemporary regimens like tamoxifen and progestins administered at high pharmacological doses (see Lønning (2004) for references). However, the lessons learned from the clinical use of these compounds, in concert with translational research evaluating their endocrine effects, provide important information to our understanding of key principles related to treatment with aromatase inhibitors.

## Endocrine principles of aromatase inhibition

In postmenopausal women, estrogens are synthesized in most of the body compartments, including the liver, muscle, connective tissue, and skin (Geisler & Lønning 2005). While one single aromatase gene exists, the gene contains at least ten different promoters (Bulun *et al*. 2003), with different promoters and ligands regulating estrogen synthesis across different tissue types (Agarwal *et al*. 1996, Clyne *et al*. 2004, Mendelson *et al*. 2005, Zhou *et al*. 2005). Notably, these promoters play a different role in benign vs malignant breast tissue; although the 1.4 promoter is the main activator in normal breast tissue, promoters II, 1.3, and 1.7 have been shown to play a role in addition to 1.4 in breast cancer tissue (Bulun *et al*. 2003). However, proteins coded for by the different promoters are similar. The aromatase is able to convert testosterone into estradiol (E_2_) and androstenedione into estrone (E_1_). While circulating androstenedione as well as testosterone in postmenopausal women is considered of adrenal origin, the ovary seems to provide a minor, albeit significant, contribution of circulating testosterone (Dowsett *et al*. 1988, Sluijmer *et al*. 1995, Couzinet *et al*. 2001). These circulating androgens are taken up by the different tissue compartments for subsequent aromatization.

Circulating androstenedione levels (4–5 nM) exceed the circulating levels of testosterone (0.5–1 nM) by a factor of about 5–10 (Geisler *et al*. 1995); in addition, the aromatase enzyme has a four- to fivefold higher affinity for androstenedione compared with testosterone (Lønning *et al*. 1990). Thus, aromatization of androstenedione into E_1_ is the major pathway of estrogen synthesis in postmenopausal women. While E_1_ is inactive by itself with respect to stimulating estrogen receptor activation, it is easily converted to E_2_ by multiple dehydrogenases (Haynes *et al*. 2010).

Considering plasma estrogen levels in postmenopausal women, plasma E_1_ (50–70 pM) exceeds E_2_ (12–20 pM) concentrations by a factor of 4–5. In addition, postmenopausal women reveal plasma concentrations of E_1_ sulfate (E_1_S) of about 4–600 pM (Geisler *et al*. 2008*b*, Lønning *et al*. 2009). To be biologically active, E_1_S has to be deconjugated prior to conversion into E_2_.

## Measuring *in vivo* aromatase inhibition

A major problem in evaluating the biochemical efficacy of aromatase inhibitors *in vivo* relates to technical difficulties measuring estrogen concentrations in the low concentration range. To achieve robust methods to assess *in vivo* aromatase inhibition and compare efficacy of different aromatase inhibitors, in collaboration with Mitch Dowsett's group, we developed a sensitive method for *in vivo* aromatization assessment based on combined ^3^H-androstenedione and ^14^C-E_1_ injections, followed by measurement of the isotope ratio in urinary estrogen metabolites (Lønning *et al*. 1989*b*, Jacobs *et al*. 1991). A formal assessment of this method revealed a sensitivity indicating an ability to detect aromatase inhibition of >99.1% in the majority of patients (Dowsett *et al*. 1995).

Using this method, we systematically classified different aromatase inhibitors (Jones *et al*. 1992, Lønning *et al*. 1991, MacNeill *et al*. 1992, 1994, 1995, Geisler *et al*. 1996*b*, 1998, 2002) based on their efficacy in inhibiting total body *in vivo* aromatization (Table 1). The findings provide three key messages; first, while it has been unclear whether the three-dimensional structure of the aromatase protein allows combined binding of a non-steroidal and a steroidal (4-hydroxyandrostenedione or exemestane) substrate-pocket binding compound, we found that adding aminoglutethimide to 4-hydroxyestrone enhanced aromatase inhibition, a finding consistent with data on plasma estrogen values with the same combined regimen (Geisler *et al*. 1996*a*). Secondly, there is a difference between ‘third-generation’ aromatase inhibitors and previous compounds regarding *in vivo* efficacy. Notably, each of the three so-called third-generation inhibitors in current use for breast cancer treatment (exemestane, anastrozole, and letrozole) causes on average >98% inhibition in individual patients. In contrast, the first- and second-generation inhibitors cause aromatase inhibition of <90%. Thirdly, this difference seems to be translated into clinically meaningful effects, as the third-generation inhibitors, in contrast to the first/second-generation compounds, have revealed clinical superiority compared with other endocrine treatment regimens (see below).

## Evaluating plasma estrogen levels in patients on treatment with aromatase inhibitors

While *in vivo* tracer injections provide the ‘gold standard’ when measuring *in vivo* aromatization and the endocrine efficacy of different aromatase inhibitors, the method is time- and source-consuming and may be applied on a limited number of patients for research purposes only. It may sometimes be necessary to determine on-treatment plasma estrogen levels in relation to treatment with different aromatase inhibitors as part of quality control.

While *in vivo* tracer studies indicate that third-generation aromatase inhibitors may inhibit total body estrogen synthesis by >98%, there are several studies reporting plasma estrogen levels to be sustained at 20–40% of pretreatment levels on therapy. As for most of these studies, clearly the assays applied did not have the sensitivity required for such a low concentration analysis. Taking normal plasma levels of E_1_ and E_2_ into account, assays with a sensitivity limit of 5–7 and 1–2 pM respectively is needed to detect >90% suppression of plasma hormone levels during treatment with an aromatase inhibitor. In addition, for steroidal compounds like exemestane, there may be cross-contamination by the drug itself or some of its metabolites, requesting pre-analytical sample purification by chromatographic methods (Johannessen *et al*. 1997). Misinterpretations due to technical problems carry a potential hazard; one example includes the use of local estrogen application for gynecological complications. Such local application leads to a modest but significant increase in plasma estrogen levels (Kendall *et al*. 2006, Wills *et al*. 2012) that may easily be overlooked with the use of less sensitive analytical methods, leading to potential erroneous conclusions regarding its safety in patients on treatment with an aromatase inhibitor.

Notably, while there is no international standardization regarding measurement of estrogens in the low concentration range, a few laboratories around the world over the years have put much effort into developing highly sensitive and specific assays for that purpose. Thus, the group headed by Mitch Dowsett in London (Dowsett *et al*. 1987, Dixon *et al*. 2008) as well as our own group (Lønning & Ekse 1995, Geisler *et al*. 2008*a*) have developed such highly sensitive assays. Using these methods, we detected plasma estrogen suppression of >90% with the third-generation aromatase inhibitors (Geisler *et al*. 1996*b*, 2008*b*, Dixon *et al*. 2008), a finding consistent with the results obtained from tracer studies.

## Normal breast tissue and intratumor estrogen levels

It has been challenged to what extent findings related to plasma estrogen suppression and total body aromatization may reflect alterations at the tumor tissue level. There are two main reasons for such concerns: first, it has been known for decades that estrogen levels are higher in tissue compared with plasma (van Landeghem *et al*. 1985). Secondly, immunostaining has revealed aromatase protein expression in breast cancer tissue (Sasano *et al*. 2005).

**Figure 1:**
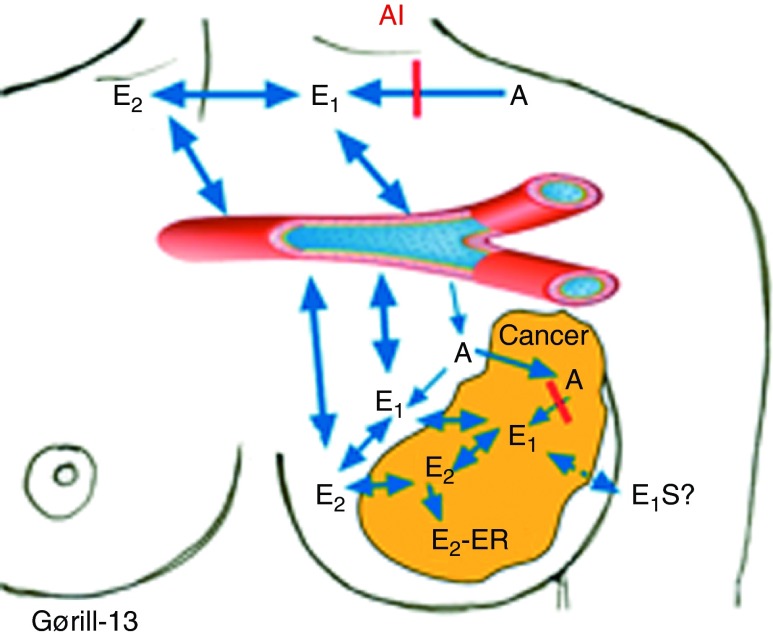
Local vs total body aromatization as a source for estrogen. There is an extensive exchange of estrone (E_1_) and estradiol (E_2_) between the plasma and the breast and breast cancer tissue due to total body aromatization, which overrules the local aromatization in the breast. Local administration of an aromatase inhibitor is therefore not a rational strategy. A, androstenedione; E_2_-ER, estradiol bound to estrogen receptor; E_1_S, estrone sulfate.

If the estrogen levels are elevated in normal as well as cancerous breast tissue due to local synthesis of estrogen, this would have significant implications in breast cancer prevention as well as therapy. First, it opens the possibility that for some tumors, lack of response could be due to inefficient tissue estrogen suppression, not detected by total body tracer studies or plasma estrogen measurement. Secondly, the fact that local aromatization is regulated by tissue-specific promoters and ligands raises the possibility of ‘targeted’ or local estrogen synthesis inhibition (Fig. 1). Such therapeutic or preventive strategies may offer great advantages, omitting unwanted side effects from systemic estrogen deprivation.

To address the topic of local estrogen production, we measured the levels of breast cancer, normal tissue, and plasma estrogens, and correlated hormone levels with the expression of hormone-modulating enzymes, including the different steroid dehydrogenases as well as aromatase and sulphokinase/sulphatase levels (Lønning *et al*. 2009, Haynes *et al*. 2010). The results have been discussed in detail elsewhere (Lønning *et al*. 2011). In brief, no correlation between intratumor estrogen levels and intratumor aromatase expression levels was found. Rather, normal breast and breast cancer tissue estrogen levels in general reflect plasma estrogen levels due to rapid equilibrium between the compartments (Fig. 1). The reason why tissue E_2_ and E_1_ levels exceed plasma concentration probably reflects the lipophilicity of these compounds; in contrast, plasma levels of E_1_S exceeds tissue concentration. In addition, there seems to be an intra-tumor conversion of E_1_ into E_2_ by local dehydrogenases. Finally, a significant amount of elevated intratumor E_2_ reflects an intratumor pool of estrogen receptor-bound hormone.

Consistent with these findings, studies by William Miller *et al*. (1998) in Edinburgh as well as our own team (Geisler *et al*. 2001, 2008*b*) have documented third-generation compounds like anastrozole and letrozole to consistently suppress intratumor estrogen levels as well. So far, there is no evidence indicating that local tumor estrogen synthesis may be a cause of therapy failure in patients on treatment with a third-generation aromatase inhibitor.

## Aromatase inhibitors in the adjuvant setting

The findings from major studies comparing third-generation aromatase inhibitors with tamoxifen for adjuvant treatment (Kaufmann *et al*. 2007, Forbes *et al*. 2008, Regan *et al*. 2011, van de Velde *et al*. 2011, Bliss *et al*. 2012, Dubsky *et al*. 2012, Boccardo *et al*. 2013) are summarized in Table 2. These studies evaluated two treatment approaches, aromatase inhibitor monotherapy or sequential treatment, where 2–3 years of tamoxifen is followed by an aromatase inhibitor. In addition, one study (BIG 1–98) also included a fourth arm; patients randomized to 2 years of letrozole, followed by 3 years with tamoxifen (Mouridsen *et al*. 2009). The rationale for the sequential approach was based on the findings from studies in the metastatic setting, revealing lack of cross-resistance between tamoxifen and aromatase inhibitors; thus, the idea was that switching from tamoxifen to an aromatase inhibitor during 5 years of adjuvant therapy may prevent acquired resistance from developing. Notably, a combined meta-analysis of these data (Dowsett *et al*. 2010) did not reveal superiority for any of the three compounds (anastrozole, letrozole, or exemestane) compared with any of the two others. The key findings, based on this meta-analysis, are summarized in the following.

Regarding the two major strategies (aromatase inhibitor monotherapy for 5 years after surgery, alternatively, tamoxifen for 2–3 years to be followed by an aromatase inhibitor for 3–2 years for a total duration of 5 years), each strategy revealed superiority compared with tamoxifen monotherapy in preventing recurrence. Among 9856 patients allocated to monotherapy with either tamoxifen or an aromatase inhibitor, following a mean duration of follow-up of 5.8 years, aromatase inhibitor monotherapy decreased relapse rate from 12.6% (for tamoxifen) to 9.6% with an aromatase inhibitor. As for survival, there was a non-significant improvement related to aromatase inhibition, but the follow-up is still too short to fully assess this end-point. Taking sequential treatment, analyzing patients from the time of randomization between continuing tamoxifen and switching to an aromatase inhibitor, the recurrence rate was reduced from 8.1 to 5% at 3 years of follow-up from randomization with a significant 0.7% reduction in breast cancer mortality among those patients receiving an aromatase inhibitor.

Based on the studies presented, aromatase inhibitors have now become standard adjuvant endocrine therapy for postmenopausal breast cancer patients. However, the data summarized above raised the question of whether aromatase inhibitor monotherapy, or sequential treatment, is the optimal strategy. In the four-arm BIG 1–98 study (Table 2), 1548 patients were randomized to tamoxifen for 2 years followed by letrozole for 3 years while 2563 patients had letrozole monotherapy (Mouridsen *et al*. 2009). At a median follow-up of 71 months from randomization, disease-free as well as overall survival were non-significantly inferior in the crossover compared with the monotherapy arm (hazard ratio (HR) of 1.05 and 1.13 respectively). Interestingly, a benefit was observed among node-positive but not among node-negative patients. In the TEAM study, tamoxifen for 2.5–3 years followed by exemestane for a total treatment duration of 5 years was compared with exemestane monotherapy (van de Velde *et al*. 2011). With a total of 9766 patients analyzed on an intention-to-treat basis and with a median follow-up of 5.1 years, no difference in disease-free survival between patients in the two arms was recorded.

As for the BIG 1–98 study, another interesting comparison was made between patients treated with letrozole upfront for 2 years followed by tamoxifen for 3 years (*n*=1540) vs letrozole monotherapy (*n*=1546). Here again, no difference in outcome between patients in the two treatment arms was recorded.

Taken together, this evidence advocates the use of aromatase inhibitors in the adjuvant treatment of postmenopausal women. However, so far, there are no strong scientific arguments in favor of either sequential or monotherapy compared with the alternative treatment strategy. Considering the cost per quality-adjusted life year gained related to each strategy, notably this depends on the relapse risk but, in addition, patient age at diagnosis (Lønning 2006).

## Duration of adjuvant therapy

While several studies have reported no additional benefit from extending tamoxifen adjuvant therapy beyond 5 years (Fisher *et al*. 1996, Tormey *et al*. 1996, Stewart *et al*. 2001), recent data from the large ATLAS study (Davies *et al*. 2012) found 10 years of tamoxifen treatment to be superior compared with 5 years of therapy. Most interestingly, while the relative ratio of recurrence was 0.90 between the two treatment arms between 5 and 9 years from diagnosis, it dropped to 0.75 after 10+ years. The fact that the benefit was delayed may explain why it was overlooked in previous studies. Furthermore, it emphasizes the importance of long-term follow-up in studies evaluating benefit from endocrine treatment.

Notably, three phase III studies have evaluated the effect of adding an aromatase inhibitor following 5 years of tamoxifen. In the MA.17 trial, patients completing 5 years of tamoxifen treatment were randomized to letrozole vs placebo (Goss *et al*. 2005); the study had to be terminated early (median follow-up of 30 months) and the patients were unblinded to treatment arm due to the extent of benefit (HR for relapse reduced to 0.58 by letrozole treatment). The results further lead to the termination of the NSABP B-33 trial (Mamounas *et al*. 2008) comparing exemestane with placebo, with a median follow-up time of 30 months, where a HR in favor of exemestane treatment of 0.68 was recorded. However, the effect was not statistically significant (*P*=0.07) due to the limited number of patients (*n*=1598) enrolled prior to early termination. Finally, the open-labeled Austrian ABCSG-6a evaluated the benefit of adding anastrozole for 3 years following 5 years of tamoxifen. At a median follow-up of 62.3 months, a benefit for extended therapy with the aromatase inhibitor was recorded (Jakesz *et al*. 2007).

While today current practice implements the use of aromatase inhibitors at an early stage during the sequence (upfront or after 2–3 years), the results from the ATLAS trial, together with the findings from studies on the extended use of aromatase inhibitors, challenge the concept of 5 years on endocrine therapy as the optimal duration. Currently, there are several studies comparing extended vs 5 years of endocrine therapy with aromatase inhibitor regimens (Table 3).

## Aromatase inhibitors in the neoadjuvant setting

Pre-surgical systemic therapy offers the benefit of down-staging tumors to allow more limited surgery (Dixon *et al*. 2009). In addition, it offers a unique setting to evaluate potential predictive factors (Lønning 2003) as well as changes in molecular parameters (Miller *et al*. 2009, Lønning & Knappskog 2013) in response to drug therapy. Several studies (Eiermann *et al*. 2001, Semiglazov *et al*. 2005, Smith *et al*. 2005, Cataliotti *et al*. 2006) have compared third-generation aromatase inhibitors to tamoxifen as primary medical therapy. Whereas letrozole (Eiermann *et al*. 2001) revealed superiority over tamoxifen, no statistically significant benefit of anastrozole compared with tamoxifen was observed in the two trials performed with this compound (Smith *et al*. 2005, Cataliotti *et al*. 2006). Regarding exemestane, one study revealed a benefit in response rate compared with tamoxifen (Semiglazov *et al*. 2005), but the number of patients was too small for statistical comparison.

During the last few years, the proliferation marker *Ki67* has become an important surrogate marker for response to endocrine therapy in the neoadjuvant setting. Comparing the percentage of *Ki67*^*+*^ proliferating cells before and after 2 weeks of endocrine therapy will indicate those patients with ER+ breast cancer that are likely to respond with tumor regression and furthermore predict their long-term outcome (Dowsett *et al*. 2005, 2007). Patients with a substantial drop in *Ki67* have been shown repeatedly to achieve the best response to such treatment (Dowsett *et al*. 2011*a*). A further benefit of *Ki67* measurement is the early identification of patients with treatment failure, as increasing Ki67 will later translate into clinical tumor progression (Dowsett *et al*. 2011*b*). In the PeriOperative Endocrine Therapy for Individualizing Care (POETIC) trial, this knowledge is expanded upon where patients with primary breast cancer are biopsied before and after 2 weeks on either a non-steroidal aromatase inhibitor or no treatment to identify novel biomarkers for response (Dowsett *et al*. 2011*b*).

## Aromatase inhibitors in metastatic disease

The role of aromatase inhibitors in metastatic disease has been reviewed elsewhere (Lønning 2004). However, the picture has changed in recent years, since today most patients with metastatic, ER-positive breast cancer have already experienced progression on adjuvant aromatase inhibition.

While previous studies revealed the superiority of aromatase inhibitors compared with tamoxifen (Mouridsen *et al*. 2001), the fact that most patients with ER+ tumors that relapse today have received an aromatase inhibitor for adjuvant therapy changes the scenario. As for patients relapsing say >1 year following termination of adjuvant therapy, re-implementation of the aromatase inhibitor may be a reasonable choice. In contrast, patients relapsing on treatment or shortly after terminating adjuvant therapy need alternative treatment options. Note that tamoxifen (Mouridsen *et al*. 2003) as well as fulvestrant (Chia *et al*. 2008) may have antitumor effects in patients where aromatase inhibitors fail, and the steroidal compound exemestane has been shown effective among patients becoming resistant to a non-steroidal aromatase inhibitor (Lønning *et al*. 2000). Interestingly, a randomized trial demonstrated a similar efficacy of fulvestrant and exemestane among patients where anastrozole or letrozole fail (Chia *et al*. 2008). Notably, most previous studies (Howell *et al*. 2002, 2004, Osborne *et al*. 2002) administered fulvestrant at a dose of 250 mg injections. While a later study revealed benefit from fulvestrant 500 mg injections compared with anastrozole in first line (Robertson *et al*. 2012), the data on fulvestrant 500 mg are not ample. Therefore, the data are insufficient to conclude that fulvestrant is superior to aromatase inhibitors, both in the first- and second-line setting. However, fulvestrant is generally well tolerated in this patient population, and the adherence to therapy is ensured by the depot i.m. injections in patients where poor compliance could be a problem.

Currently, there is no general consensus regarding optimal sequencing of endocrine therapy for metastatic breast cancer. However, for patients with metastatic ER+ breast cancer, it remains important to extend endocrine treatment for as long as their disease responds, prior to implementing chemotherapy.

## Side effects of aromatase inhibitors

Estrogens play a key role in many physiological processes other than reproduction. Thus, aromatase knock-out mice reveal multiple metabolic defects (Jones *et al*. 2001), and aromatase deficiency due to germline mutations causes osteopenia as well as metabolic disturbances in both genders (Morishima *et al*. 1995, Rochira & Carani 2009). Thus, a major concern with respect to aromatase inhibition in early breast cancer has been the enhanced bone loss as well as disturbances in lipid metabolism, which could increase the risk of cardiovascular diseases.

Osteoporosis is a major health threat to the aging female population in most countries. Osteoporotic fractures are associated with a significant morbidity and excess mortality (Johnell *et al*. 2004). The lifetime risk for a hip fracture among European and USA Caucasian females is in the range of 15–20%. In some countries, like in Scandinavia, it may exceed 25% (Kanis *et al*. 2002). It is now well established that all aromatase inhibitors moderately enhance bone loss. However, most studies have addressed the effect of aromatase inhibitors on bone loss in phase III studies comparing efficacy and side effects to tamoxifen (Coleman *et al*. 2007, Eastell *et al*. 2008), the second expressing anabolic effects on bone metabolism in postmenopausal women (Powles *et al*. 1996). The effects of exemestane (Lønning *et al*. 2005) as well as letrozole (Perez *et al*. 2006), however, on bone metabolism have also been compared with placebo, revealing a moderate loss in bone density. Notably, while ongoing treatment with an aromatase inhibitor is associated with increased bone fracture rate (Coates *et al*. 2007, Coombes *et al*. 2007, Eastell *et al*. 2008) in comparison with tamoxifen, any detrimental effects of aromatase inhibitors on bone metabolism disappear upon terminating the drug (Geisler *et al*. 2006, Eastell *et al*. 2008). With the encouraging results from the Austrian Breast Cancer Group, revealing that zoledronic acid may completely prevent aggravated bone loss, even among premenopausal women exposed to ovarian ablation and anastrozole in concert (Gnant *et al*. 2008), detrimental effects on bone metabolism may be fully preventable.

Another major concern has been with respect to detrimental effects of estrogen suppression on lipid metabolism (Engan *et al*. 1995) and homocysteine levels (Anker *et al*. 1995) that could lead to an increased risk of cardiovascular disease. As for the latter, contrary to previous claims, recent evidence suggest that plasma homocysteine may not be a major risk factor with respect to cardiovascular disease after all (Bonaa *et al*. 2006). For decades, estrogen replacement therapy was believed to protect against cardiovascular events in postmenopausal women. However, while hormone replacement therapy slightly elevates HDL-cholesterol levels, this effect seems not to translate into a reduced risk of cardiovascular disease (Trial 1995, Hulley *et al*. 1998, Alexander *et al*. 2001, Manson *et al*. 2003, Anderson *et al*. 2004). Considering the effects of aromatase inhibitors on plasma lipid levels, studies conducted on non-fasting subjects as well as studies on patients with metastatic disease, often suffering from metabolic disturbances, are subject to multiple confounding variables (see Lønning & Geisler (2008) for discussion and references). In the two studies evaluating the effects of an aromatase inhibitor vs placebo in early disease, both exemestane (Lønning *et al*. 2005) as well as letrozole (Wasan *et al*. 2005) had minor effects on plasma lipid levels. Considering cardiovascular events in the phase III trials comparing aromatase inhibitors to tamoxifen (Table 4), there is no substantial evidence suggesting detrimental effects of aromatase inhibitors with respect to cardiovascular morbidity and mortality in early breast cancer (Jakesz *et al*. 2005, Boccardo *et al*. 2006, 2013, Kaufmann *et al*. 2007, Forbes *et al*. 2008, Colleoni *et al*. 2011, van de Velde *et al*. 2011, Bliss *et al*. 2012, Dubsky *et al*. 2012).

A third type of side effects now receiving more attention is musculoskeletal: joint pain and stiffness, including carpal tunnel syndrome (Morales *et al*. 2007, Nishihori *et al*. 2008, Dizdar *et al*. 2009, Sestak *et al*. 2009, Mieog *et al*. 2012). While most patients have moderate disturbances, there is evidence that probably 20% of the patient population do not adhere to prescribed therapy with aromatase inhibitors (Partridge *et al*. 2008), and musculoskeletal and joint pain may be responsible for at least 50% of these withdrawals (Dent *et al*. 2007). Notably, Belgian investigators reported synovial deposits detectable by magnetic resonance imaging (MRI) scans among patients suffering from tendon and joint pain (Morales *et al*. 2008). For these patients, tamoxifen, or probably fulvestrant, may be considered as alternative treatment options. Interestingly, it seems that certain single nucleotide polymorphisms are associated with the musculoskeletal side effects of aromatase inhibitors, related to the expression of interleukin 17 receptor A (Ingle *et al*. 2010). Such analysis might allow us to identify upfront patients in the future who will not tolerate aromatase inhibitors and should have another endocrine treatment.

## Aromatase inhibitors and obesity

Obesity is associated with significantly elevated risk of breast cancer (Morimoto *et al*. 2002, Key *et al*. 2003) as well as a poor prognosis among postmenopausal breast cancer patients (Protani *et al*. 2010, Kwan *et al*. 2012). While the mechanisms are incompletely understood, the fact that obesity has been associated with elevated levels of E_2_ in postmenopausal women (Meldrum *et al*. 1981, Poortman *et al*. 1981), as well as recent findings indicating that obesity may not influence outcome in triple-negative breast cancers (Dawood *et al*. 2012), indicate that elevated E_2_ levels may be (at least partly) responsible for these effects. However, others (Niraula *et al*. 2012) have argued that obesity may confer a poor prognosis, independent of estrogen receptor levels and menopausal status.

Recent data have thrown concern over the efficacy of aromatase inhibitors among obese individuals. The Austrian ABCSG-12 trial randomized premenopausal breast cancer patients in a 2×2 trial design to either treatment with goserelin plus tamoxifen+/−zoledronic acid or goserelin plus anastrozole+/−zoledronic acid. Recently, Pfeiler *et al*. (2011) reported overweight (BMI >25) to be associated with an enhanced relapse rate within the group of patients treated with anastrozole. In contrast, no detrimental effect of obesity was observed among patients treated with tamoxifen. Moreover, overweight individuals treated with anastrozole had a 50% increased risk of a relapse but a threefold increased risk of death compared with overweight patients on tamoxifen treatment. Analyzing the ATAC study, Sestak *et al*. (2010) found women with a BMI >35 to have a poor prognosis compared with lean women, independent of treatment arm (anastrozole or tamoxifen). However, there was a non-significant trend indicating a reduced benefit of anastrozole compared with tamoxifen among obese individuals. In contrast, analyzing data from the BIG 1–98 study, Ewertz *et al*. (2012) found the benefit of letrozole compared with tamoxifen to be independent of BMI value. The question of whether obese patients on treatment with aromatase inhibitors express elevated plasma estrogen levels compared with individuals with a normal BMI is a current issue of controversy (Diorio *et al*. 2012, Folkerd *et al*. 2012).

Taken together, data at this stage do not advocate that aromatase inhibitors should be avoided among overweight patients. Importantly, there are several potential explanations to the effects observed in the Austrian trial. If the problem is failure on goserelin among obese individuals, this may be predicted to have little (if any) effect on patients treated with tamoxifen in concert, while it could be detrimental to the effect of anastrozole; it is well known that aromatase inhibitors may not prevent ovarian estrogen synthesis among pre- and perimenopausal women (Geisler & Lønning 2005). At this stage, it may be wise to do regular plasma hormone assessment among overweight and obese premenopausal women having goserelin treatment independent of whether they receive concomitant treatment with an aromatase inhibitor or not. As for postmenopausal women receiving aromatase inhibitor monotherapy, notably, the detrimental effect of body weight observed on anastrozole treatment related to patients with a BMI >35. Further, the fact that no detrimental effect of obesity was recorded for patients treated with letrozole should be underlined. *In vivo* studies demonstrated letrozole to be significantly more potent than anastrozole in inhibiting total body *in vivo* aromatization (Geisler *et al*. 2002) and suppressing breast cancer tissue estrogen levels (Geisler *et al*. 2008*b*). In summary, while there may be some uncertainty related to the use of anastrozole among obese patients, data so far (at least with respect to letrozole) seem reassuring.

## Molecular markers predicting benefit to aromatase inhibitors compared with tamoxifen?

While only about 10% of ER-positive tumors overexpress HER-2 (Sørlie *et al*. 2001, Penault-Llorca *et al*. 2009), notably about 50% of all HER-2-amplified tumors are positive for ER expression, although at low or moderate levels (Untch *et al*. 2008, Penault-Llorca *et al*. 2009). Comparing letrozole with tamoxifen as pre-surgical therapy, Ellis *et al*. (2001) reported a particular superiority for letrozole over tamoxifen in tumors overexpressing either HER-1 or HER-2. Moreover, they reported letrozole to provide a particular benefit compared with tamoxifen for patients with tumors revealing a moderate Allred ER score. However, these findings have not been reproduced in the phase III adjuvant studies. Thus, data from the TransATAC, comparing anastrozole with tamoxifen (Dowsett *et al*. 2008), as well as the BIG 1–98, comparing letrozole with tamoxifen (Rasmussen *et al*. 2008), revealed a higher relapse rate for patients with HER-2-positive vs HER-2-negative tumors in the aromatase inhibitor as well as in the tamoxifen-treated arm. The relative benefit for the aromatase inhibitor over tamoxifen, however, was similar in both patient groups. Similarly, separating patients into quartiles based on ER expression status, Dowsett *et al*. (2008), found the relative benefit from anastrozole over tamoxifen to be independent of ER expression status.

**Figure 2:**
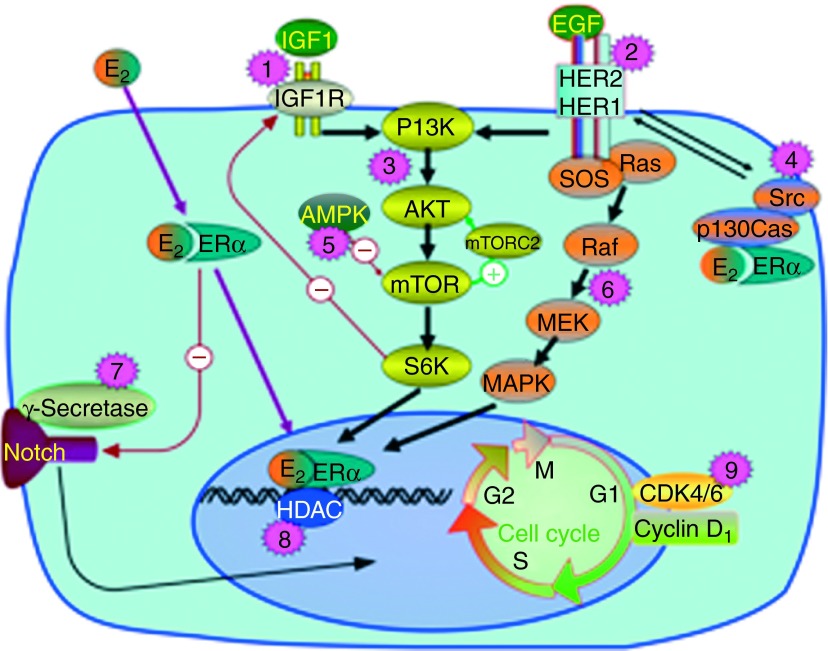
Signaling mechanisms important for endocrine resistance and which are currently targeted in clinical trials, combined with aromatase inhibitors. (1) IGF1 or IGF1R neutralizing antibodies (AMG-479 in study NCT00626106 a.o.). (2) HER-2 blocking therapy (trastuzumab emtansine in study NCT01745965 a.o.). (3) Inhibitors of PI3K, Akt, and/or mTOR pathway (everolimus in study NCT01698918 a.o.). (4) Src inhibitors (dasatinib in study NCT00696072 a.o.). (5) AMPK activator (metformin in study NCT01654185 a.o.). (6) Inhibitors of Ras-Raf-MEK-MAPK pathway (MEK inhibitor AZD6244, combined with fulvestrant after progression on aromatase inhibitor in NCT01160718). (7) Gamma secretase inhibitor (RO4929097 in study NCT01208441 a.o.). (8) HDAC inhibitors (vorinostat in study NCT01153672 a.o.). (9) CDK4/6 inhibitor (PD0332991 in study NCT01740427 a.o.).

Notably, adding either trastuzumab (Kaufman *et al*. 2009) or lapatinib (Johnston *et al*. 2009) to treatment with an aromatase inhibitor in patients with ER+/HER-2+ metastatic breast cancer improves time to progression. Whether this relates to reversal of endocrine resistance or, simply, to different treatment options administered in concert is not known. However, experimental studies have revealed cross talk between HER-2 and ER signaling mediated via both the PI3K-Akt-mTOR and the Ras-Raf-MEK-MAPK pathways (Campbell *et al*. 2001, Knowlden *et al*. 2003, Jordan *et al*. 2004, Jelovac *et al*. 2005). Interestingly, the study by Johnston *et al*. (2009) also included patients with ER-positive tumors harboring a normal HER-2 status. While no benefit for lapatinib was recorded among HER-2-negative tumors on an intention-to-treat basis, a pre-defined analysis revealed superiority for lapatinib in a subgroup of HER-2 non-amplified tumors with an early relapse on tamoxifen. Potential interactions between the HER-2 and ER pathways should be further examined. Accordingly, we found that primary treatment with aromatase inhibitors may increase tumor HER-2 levels in non-amplified tumors (Flageng *et al*. 2009). Currently, the GCC 0901 study examines the effect of adding the mTOR inhibitor everolimus to patients progressing on letrozole and lapatinib in concert (ClinicalTrials.gov #NCT01499160). However, in HER-2 overexpressing breast cancer, PI3K signaling inhibition leads to increased HER-2-mediated ERK activation, pointing to yet another important growth-promoting signaling axis, the Ras-Raf-MEK-ERK pathway (Fig. 2), and the potential need for adding for instance MEK inhibitors in certain patient subgroups (Serra *et al*. 2011).

## Resistance toward treatment with aromatase inhibitors: current observations related to targeted therapy

As mentioned above, there is increasing evidence indicating that cross talk between the estrogen receptor pathway and several other growth-controlling pathways may cause resistance to aromatase inhibitors (Miller *et al*. 2011, Sabnis & Brodie 2011). Accordingly, a large number of clinical trials are currently conducted to assess whether various signal transduction inhibitors can augment the efficacy of aromatase inhibitors in postmenopausal patients with breast cancer. These include inhibitors of the PI3K-Akt-mTOR and Ras-Raf-MEK-MAPK pathways, insulin-like growth factor 1 (IGF1) receptor (IGF1R), gamma secretase/Notch, cyclin-dependent kinase 4/6 (CDK4/6), histone deacetylase (HDAC), and Src/Abl a.o. (Fig. 2), as elaborated on elsewhere (Fedele *et al*. 2012). For instance, adding the CDK4/6 inhibitor PD0332991 to letrozole in the first-line treatment of patients with ER+ metastatic breast cancer increased PFS from 7.5 to 26.1 months in a phase II study with 165 patients (Finn *et al*. 2012).

The PI3K-Akt-mTOR signaling pathway has come up as a major resistance pathway in endocrine resistance, including resistance to aromatase inhibitors (Miller *et al*. 2011, Villarreal-Garza *et al*. 2012). Activating mutation in *PIK3CA*, the gene encoding the p100α subunit of the PI3K protein, is present in 28–47% of ER-positive breast cancers, and is the single gene most frequently mutated in this disease (Miller *et al*. 2011). Currently, numerous studies are conducted where drugs targeting PI3K, Akt, and/or mTOR are tested in combination with aromatase inhibitors (Baselga 2011). Recently, two studies revealed that the mTOR inhibitor everolimus significantly improves time to progression in patients with ER-positive metastatic breast cancer undergoing endocrine therapy. The BOLERO-2 study randomized a total of 742 patients developing resistance to a non-steroidal aromatase inhibitor at a 2:1 ratio between exemestane plus everolimus vs exemestane monotherapy; here, median time to progression was extended from 4.1 to 10.6 months (Baselga *et al*. 2012). The effect seems not to be limited to the use of aromatase inhibitors; in a smaller study, Bachelot *et al*. (2012) found everolimus to improve time to progression among patients treated with tamoxifen. While this study indicated a benefit among patients with acquired resistance only, the number of patients was too low to allow for any definite conclusion.

However, there are results at variance. In a recent study, Wolff *et al*. (2013) found only a non-significant and modest benefit from adding another mTOR inhibitor temsirolimus to letrozole in aromatase inhibitor-naïve patients, independent of previous adjuvant tamoxifen therapy. While these results, like the findings by Bachelot *et al*. (2012), may indicate an effect of mTOR inhibition on acquired but not primary drug resistance across different compounds, more data are needed to draw any final conclusion. This issue will be addressed further in the BOLERO-4 phase II study, where 200 patients with metastatic breast cancer will be treated with everolimus and letrozole in the first-line setting (NCT01698918).

Furthermore, it should be recalled that the PI3K-Akt-mTOR pathway is involved in chemotherapy resistance as well (Lønning & Knappskog 2013). Additionally, mTOR inhibitors have revealed antitumor efficacy across other tumor forms, indicating that they exhibit anti-tumor efficacy by themselves, not only as adjuvants to other cancer therapies (Motzer *et al*. 2010, Yao *et al*. 2010).

An interesting extension of the combined mTOR inhibitor/endocrine therapy trials in breast cancer is what to do when the treatment fails. In the BELLE-3 trial, a pan-PI3K inhibitor BKM120 or placebo is combined with fulvestrant in patients with advanced breast cancer who have progressed on an mTOR inhibitor plus endocrine therapy (NCT01633060). This trial will address whether upstream inhibition of PI3K is worthwhile in patients where an mTOR inhibitor fails. Moreover, rapamycin analogs (rapalogs), which are the kind of mTOR inhibiting drugs currently used in the clinic, mainly inhibit mTORC1 but not the mTORC2 complex (Jacinto *et al*. 2004, Villarreal-Garza *et al*. 2012), and the consequence of this is ongoing Akt activation by mTORC2 during rapalog administration (Fig. 2). Second-generation mTOR inhibitors, like PP242 and CC-223, inhibit both mTORC1 and mTORC2 (Janes *et al*. 2010, Weigelt *et al*. 2011); however, the potential benefit from this additional effect remains to be evaluated. Another important question is whether to keep the mTOR inhibitor beyond progression. In a planned phase II study by the German Breast Group, patients who exhibit progression of their disease on exemestane and everolimus change their endocrine treatment whereas everolimus is kept as the backbone (NCT01773460).

Another possible resistance mechanism has also been identified where aromatase inhibitors fail due to platelet-derived growth factor receptor (PDGFR)/Abl signaling upregulation (Weigel *et al*. 2013). Src is a downstream hub for various signaling pathways, like EGFR and HER-2, and a potential signaling link between non-genomic ER and HER-2 via p130Cas, which might be involved in resistance to endocrine therapy (Cabodi *et al*. 2004, Fox *et al*. 2009, Vallabhaneni *et al*. 2011). Src, Abl, and PDGFR can be inhibited by the oral small molecule inhibitor dasatinib, which is already registered for use in chronic myeloid leukemia with BCR-ABL mutation, resistant to imatinib. Combination clinical trials of dasatinib and aromatase inhibitors are currently ongoing.

Another interesting observation regarding resistance to aromatase inhibitors relates to the *in vitro* phenomenon of ‘estrogen hypersensitivity’. Breast cancer cells that have grown under long-term estrogen deprivation (LTED) become extremely sensitive to estrogen (Masamura *et al*. 1995, Santen *et al*. 2005). Whereas estrogen at high concentrations normally stimulates cell growth, it becomes cytotoxic in LTED cells (Lønning *et al*. 2001). While the exact mechanism causing LTED has not been fully elucidated, upregulation of the ERα, in addition to the PI3K-mTOR, and MAPK pathways has been shown to occur in LTED cells. Taking the concept of ‘estrogen hypersensitivity’ to the clinic, we demonstrated that estrogen in high doses can be used therapeutically in ER-positive breast cancer with acquired resistance to aromatase inhibitors (Lønning *et al*. 2001), a finding subsequently confirmed by Ellis *et al*. (2009).

## Future aspects on aromatase inhibition and issues that remain to be settled

While aromatase inhibitors have become the standard therapy for ER+ postmenopausal breast cancer, several issues remain to be settled. A current issue relates to the optimal duration of therapy. The next decade will address the question of how long aromatase inhibitors should be administered to derive the maximum benefit in the adjuvant setting. In this respect, the initial results of the ongoing MA.17 extension trial and NSABP B-42 study are expected in 2015. This primarily relates to therapeutic efficacy, but long-term toxicity is another important aspect in the adjuvant setting. Whereas the issue of toxicity has been thoroughly addressed with respect to 5 years of treatment, potential side effects related to extended therapy need to be carefully monitored.

A key issue with respect to treatment with aromatase inhibitors, like most other cancer compounds (Lønning & Knappskog 2013), relates to the design of targeted strategies to prevent drug resistance *in vivo*. Currently, studies, like the POETIC trial, have been designed to specifically address this issue. We have an emerging understanding of which signaling pathways are involved in endocrine resistance and an increasing number of signal transduction inhibitors to target these pathways. However, the challenge will be which drugs to pick, how to combine them, and how they will be tolerated with respect to side effects (for the patient) and economic cost (for society).

Clearly, large phase III trials cannot be used when dozens of deregulated signaling pathways are to be targeted simultaneously. Importantly, new technology, such as ‘next-generation deep sequencing’, is becoming increasingly efficient and affordable, which, in the near future, will allow an increasing number of research laboratories to conduct complete exome sequencing of individual tumors. Such extensive understanding of each tumor's genomic profile, merged with biological evidence linking disturbances in defined growth factor pathways to endocrine resistance, has the potential to optimize endocrine therapy dramatically in the future. In studies, such as the ongoing SHIVA study in France (NCT01771458), the molecular profile of each individual cancer is used to design targeted therapy, as opposed to conventional therapy, to compare the old and new school in oncology. The therapeutic potential of such strategies will be answered shortly.

## Figures and Tables

**Table 1:** Maximum inhibition of total body aromatization obtained with previously and currently used aromatase inhibitors

	**Generation**	**Maximum inhibition** (%)	**References**
Rogletimide	First	74	MacNeill *et al*. (1992)
Aminoglutethimide	First	91	MacNeill *et al*. (1992)
Aminoglutethimide+formestane	First/second	94	MacNeill *et al*. (1994)
Formestane (oral)	Second	70	MacNeill *et al*. (1995)
Fadrozole	Second	93	Lønning *et al*. (1991)
Formestane (i.m.)	Second	92	Jones *et al*. (1992)
Letrozole	Third	99.1	Dowsett *et al*. (1995) and Geisler *et al*. (2002)
Anastrozole	Third	98.1	Geisler *et al*. (1996*b*, 2002)
Exemestane	Third	97.9	Geisler *et al*. (1998)

**Table 2:** Results of the major adjuvant studies comparing third-generation aromatase inhibitors and tamoxifen

**No. of patients**	**Drug**	**Survival**		**Follow-up**	**References**
DFS	OS
ATAC					
3116	T	0.90^a^	0.97	100 months	Forbes *et al*. (2008)
3125	A				
3125	T→A^b^				
BIG 1–98					
2459	T	0.86^a^	0.87^a^	8.1 years	Regan *et al*. (2011)
2463	L				
1545	L→T	1.06^c^	0.97^d^		
1548	T→L	1.07^c^	1.10^d^		
ABCSG-8					
1849	T	0.80^d^	0.87^d^	60 months	Dubsky *et al*. (2012)
1865	T→A				
ARNO 95					
490	T	0.66^e^	0.53^e^	30.1 months	Kaufmann *et al*. (2007)
489	T→A				
ITA					
225	T	0.64^e^	0.79^d^	128 months	Boccardo *et al*. (2013)
223	T→A				
IES					
2305	T	0.81^e^	0.86^e^	91 months	Bliss *et al*. (2012)
2294	T→E				
TEAM					
4868	T→E	0.97^d^	1.00^d^	5.1 years	van de Velde *et al*. (2011)
4898	E				

T, tamoxifen; A, anastrozole; L, letrozole; E, exemestane.

a Significant difference, in favor of aromatase inhibitor.

b This arm was discontinued after the initial efficacy analysis showed no benefit over tamoxifen alone. No long-term follow-up for this group.

c No significant difference, compared with letrozole.

d No significant difference.

e Significant difference, in favor of sequence tamoxifen–aromatase inhibitor.

**Table 3:** Ongoing studies comparing extended vs 5 years of endocrine therapy with aromatase inhibitor regimens

	**No. of patients**	**Years 1–5**	**Years 5–10**	**Years 10–15**	**Results expected**	**ClinicalTrials.gov**
MA.17 extension trial	1918	T	L or placebo	L or placebo^a^	2015	NCT00754845
NSABP B-42	3966	AI or T→AI	L or placebo	–	2015	NCT00382070

a The study compares an additional 5 years of letrozole/placebo after completing 5 years of tamoxifen and 5 years of letrozole for patients in the original MA.17 study, or letrozole/placebo years 5–10 for patients that previously got an aromatase inhibitor years 1–5.

**Table 4:** Cardiovascular events in adjuvant phase III trials comparing aromatase inhibitors to tamoxifen

**Patients**	**Drug**	**Cardiovascular events**			**Follow-up**	**References**
Cardiac AE^a^	IHD^b^	Deaths^c^
ATAC						
3116	T	3.4	0.27^d^	66	100 months	Forbes *et al*. (2008)
3125	A	4.1	0.27^d^	67		
BIG 1–98						
2447	T	6.2	2.0	7	74 months	Colleoni *et al*. (2011)
2448	L	6.9	2.8	10		
ABCSG-8						
1849	T	4.4^e^	<1.0^d^	NR	60 months	Jakesz *et al*. (2005) and Dubsky *et al*. (2012)
1865	T→A	4.2^e^	<1.0^d^	NR		
ARNO 95						
452	T	NR	0.9	16^f^	30.1 months	Kaufmann *et al*. (2007)
445	T→A	NR	2.0	11^f^		
ITA						
225	T	6.2	NR	11^f^	128 months	Boccardo *et al*. (2006, 2013)
223	T→A	7.6	NR	12^f^		
IES						
2036	T	10.4	4.6	20	91 months	Bliss *et al*. (2012)
2105	T→E	12.3	6.0	22		
TEAM						
4814	T→E	6.4	1.0	28	5.1 years	van de Velde *et al*. (2011)
4852	E	8.1^g^	2.0	43		

T, tamoxifen; A, anastrozole; L, letrozole; E, exemestane; NR, not reported.

a Any grade cardiovascular adverse event (%), while on therapy or within 30 days of drug discontinuation. No significant difference between groups unless clearly marked.

b Ischemic heart disease.

c Number of deaths from cardiovascular causes.

d Both cardiovascular and thromboembolic events.

e Myocardial infarction only.

f All non-cancer-related deaths.

g Significantly more cases of cardiac failure in exemestane alone group.

## References

[bib1] Agarwal VR, Bulun SE, Leitch M, Rohrich R, Simpson ER (1996). Use of alternative promoters to express the aromatase cytochrome p450 (CYP19) gene in breast adipose tissues of cancer-free and breast cancer patients. Journal of Clinical Endocrinology and Metabolism.

[bib2] Alexander KP, Newby LK, Hellkamp AS, Harrington RA, Peterson ED, Kopecky S, Langer A, O'Gara P, O'Connor CM, Daly RN (2001). Initiation of hormone replacement therapy after acute myocardial infarction is associated with more cardiac events during follow-up. Journal of the American College of Cardiology.

[bib3] Anderson GL, Limacher M, Assaf AR, Bassford T, Beresford SAA, Black H, Bonds D, Brunner R, Brzyski R, Caan B (2004). Effects of conjugated, equine estrogen in postmenopausal women with hysterectomy – The Women's Health Initiative Randomized Controlled trial. Journal of the American Medical Association.

[bib4] Anker G, Lønning PE, Ueland PM, Refsum H, Lien EA (1995). Plasma levels of the atherogenic amino acid homocysteine in post-menopausal women with breast cancer treated with tamoxifen. International Journal of Cancer.

[bib5] Bachelot T, Bourgier C, Cropet C, Ray-Coquard I, Ferrero JM, Freyer G, Abadie-Lacourtoisie S, Eymard JC, Debled M, Spaëth D (2012). Randomized phase II trial of everolimus in combination with tamoxifen in patients with hormone receptor-positive, human epidermal growth factor receptor 2-negative metastatic breast cancer with prior exposure to aromatase inhibitors: a GINECO study. Journal of Clinical Oncology.

[bib6] Baselga J (2011). Targeting the phosphoinositide-3 (PI3) kinase pathway in breast cancer. Oncologist.

[bib7] Baselga J, Campone M, Piccart M, Burris HA, Rugo HS, Sahmoud T, Noguchi S, Gnant M, Pritchard KI, Lebrun F (2012). Everolimus in postmenopausal hormone-receptor-positive advanced breast cancer. New England Journal of Medicine.

[bib8] Beatson GT (1896). On the treatment of inoperable cases of carcinoma of the mamma. Suggestions for a new method of treatment with illustrative cases. Lancet.

[bib9] Bliss JM, Kilburn LS, Coleman RE, Forbes JF, Coates AS, Jones SE, Jassem J, Delozier T, Andersen J, Paridaens R (2012). Disease-related outcomes with long-term follow-up: an updated analysis of the Intergroup Exemestane Study. Journal of Clinical Oncology.

[bib10] Boccardo F, Rubagotti A, Guglielmini P, Fini A, Paladini G, Mesiti M, Rinaldini M, Scali S, Porpiglia M, Benedetto C (2006). Switching to anastrozole versus continued tamoxifen treatment of early breast cancer. Updated results of the Italian Tamoxifen Anastrozole (ITA) trial. Annals of Oncology.

[bib11] Boccardo F, Guglielmini P, Bordonaro R, Fini A, Massidda B, Porpiglia M, Roagna R, Serra P, Orzalesi L, Ucci G (2013). Switching to anastrozole versus continued tamoxifen treatment of early breast cancer: long term results of the Italian Tamoxifen Anastrozole trial. European Journal of Cancer.

[bib12] Bonaa KH, Njolstad I, Ueland PM, Schirmer H, Tverdal A, Steigen T, Wang H, Nordrehaug JE, Arnesen E, Rasmussen K (2006). Homocysteine lowering and cardiovascular events after acute myocardial infarction. New England Journal of Medicine.

[bib13] Brodie AMH, Schwarzel WC, Shaikh AA, Brodie HJ (1977). The effect of an aromatase inhibitor, 4-hydroxy-androstene-3,17-dione, on estrogen-dependent processes in reproduction and breast cancer. Endocrinology.

[bib14] Brodie AMH, Garrett WM, Hendrickson JR, Tsai-Morris CH, Williams JG (1983). Aromatase inhibitors, their pharmacology and application. Journal of Steroid Biochemistry.

[bib15] Bulun SE, Sebastian S, Takayama K, Suzuki T, Sasano H, Shozu M (2003). The human CYP19 (aromatase P450) gene: update on physiologic roles and genomic organization of promoters. Journal of Steroid Biochemistry and Molecular Biology.

[bib16] Cabodi S, Moro L, Baj G, Smeriglio M, Di Stefano P, Gippone S, Surico N, Silengo L, Turco E, Tarone G (2004). p130Cas interacts with estrogen receptor α and modulates non-genomic estrogen signaling in breast cancer cells. Journal of Cell Science.

[bib17] Campbell RA, Bhat-Nakshatri P, Patel NM, Constantinidou D, Ali S, Nakshatri H (2001). Phosphatidylinositol 3-kinase/AKT-mediated activation of estrogen receptor α – a new model for anti-estrogen resistance. Journal of Biological Chemistry.

[bib18] Cash R, Brough AJ, Cohen MNP, Satoh PS (1967). Aminoglutethimide (Elipten-Ciba) is an inhibitor of adrenal steroidogenesis: mechanism of action and therapeutic trial. Journal of Clinical Endocrinology and Metabolism.

[bib19] Cataliotti L, Buzdar AU, Noguchi S, Bines J, Takatsuka Y, Petrakova K, Dube P, de Oliveira CT (2006). Comparison of anastrozole versus tamoxifen as preoperative therapy in postmenopausal women with hormone receptor-positive breast cancer – The Pre-Operative ‘Arimidex’ Compared to Tamoxilen (PROAC7) trial. Cancer.

[bib20] Chia S, Gradishar W, Mauriac L, Bines J, Amant F, Federico M, Fein L, Romieu G, Buzdar A, Robertson JF (2008). Double-blind, randomized placebo controlled trial of fulvestrant compared with exemestane after prior nonsteroidal aromatase inhibitor therapy in postmenopausal women with hormone receptor-positive, advanced breast cancer: results from EFECT. Journal of Clinical Oncology.

[bib21] Clyne CD, Kovacic A, Speed CJ, Zhou J, Pezzi V, Simpson ER (2004). Regulation of aromatase expression by the nuclear receptor LRH-1 in adipose tissue. Molecular and Cellular Endocrinology.

[bib22] Coates AS, Keshaviah A, Thurlimann B, Mouridsen H, Mauriac L, Forbes JF, Paridaens R, Castiglione-Gertsch M, Gelber RD, Colleoni M (2007). Five years of letrozole compared with tamoxifen as initial adjuvant therapy for postmenopausal women with endocrine-responsive early breast cancer: update of study BIG 1–98. Journal of Clinical Oncology.

[bib23] Coleman R, Banks L, Girgis S, Kilburn L, Vrdoljak E, Fox J, Cawthorn SJ, Patel A, Snowdon CF, Hall E (2007). Skeletal effects of exemestane on bone-mineral density bone biomarkers and fracture incidence in postmenopausal women with early breast cancer participating in the Intergroup Exemestane Study (IES): a randomised controlled study. Lancet Oncology.

[bib24] Colleoni M, Giobbie-Hurder A, Regan MM, Thurlimann B, Mouridsen H, Mauriac L, Mauriac L, Forbes JF, Paridaens R, Láng I (2011). Analyses adjusting for selective crossover show improved overall survival with adjuvant letrozole compared with tamoxifen in the BIG 1–98 Study. Journal of Clinical Oncology.

[bib25] Coombes RC, Goss P, Dowsett M, Gazet J-C, Brodie A (1984). 4-Hydroxyandrostenedione in treatment of postmenopausal patients with advanced breast cancer. Lancet.

[bib26] Coombes RC, Kilburn LS, Snowdon CF, Paridaens R, Coleman RE, Jones SE, Jassem J, Van de Velde CJ, Delozier T, Alvarez I (2007). Survival and safety of exemestane versus tamoxifen after 2–3 years' tamoxifen treatment (Intergroup Exemestane Study): a randomised controlled trial. Lancet.

[bib27] Couzinet B, Meduri G, Lecce M, Young J, Brailly S, Loosfelt H, Milgrom E, Schaison G (2001). The postmenopausal ovary is not a major androgen-producing gland. Journal of Clinical Endocrinology and Metabolism.

[bib28] Davies C, Pan HC, Godwin J, Gray R, Peto R, Collaboratives A (2012). ATLAS – 10 v 5 years of adjuvant tamoxifen (TAM) in ER+ disease; effects on outcome in the first and second decade after diagnosis. Cancer Research.

[bib29] Dawood S, Lei XD, Litton JK, Buchholz TA, Hortobagyi GN, Gonzalez-Angulo AM (2012). Impact of body mass index on survival outcome among women with early stage triple-negative breast cancer. Clinical Breast Cancer.

[bib30] Dent S, Hopkins S, Di Valentin T, Verreault J, Vandermeer L, Verma S (2007). Adjuvant aromatase inhibitors in early breast cancer – toxicity and adherence. Important observations in clinical practice. Breast Cancer Research and Treatment.

[bib31] Diorio C, Lemieux J, Provencher L, Hogue JC, Vachon E (2012). Aromatase inhibitors in obese breast cancer patients are not associated with increased plasma estradiol levels. Breast Cancer Research and Treatment.

[bib32] Dixon JM, Renshaw L, Young O, Murray J, Macaskill EJ, McHugh M, Folkerd E, Cameron DA, A'Hern RP, Dowsett M (2008). Letrozole suppresses plasma estradiol and estrone sulphate more completely than anastrozole in postmenopausal women with breast cancer. Journal of Clinical Oncology.

[bib33] Dixon JM, Renshaw L, Macaskill EJ, Young O, Murray J, Cameron D, Kerr GR, Evans DB, Miller WR (2009). Increase in response rate by prolonged treatment with neoadjuvant letrozole. Breast Cancer Research and Treatment.

[bib34] Dizdar O, Ozcakar L, Malas FU, Harputluoglu H, Bulut N, Aksoy S, Ozisik Y, Altundag K (2009). Sonographic and electrodiagnostic evaluations in patients with aromatase inhibitor-related arthralgia. Journal of Clinical Oncology.

[bib35] Dowsett M, Goss PE, Powles TJ, Hutchinson G, Brodie AMH, Jeffcoate SL, Coombes RC (1987). Use of the aromatase inhibitor 4-hydroxyandrostenedione in postmenopausal breast cancer: optimization of therapeutic dose and route. Cancer Research.

[bib36] Dowsett M, Cantwell B, Lal A, Jeffcoate SL, Harris AL (1988). Suppression of postmenopausal ovarian steroidogenesis with the luteinizing hormone-releasing hormone agonist goserelin. Journal of Clinical Endocrinology and Metabolism.

[bib37] Dowsett M, Jones A, Johnston SRD, Jacobs S, Trunet P, Smith IE (1995). *In vivo* measurement of aromatase inhibition by letrozole (CGS 20267) in post menopausal patients with breast cancer. Clinical Cancer Research.

[bib38] Dowsett M, Smith IE, Ebbs SR, Dixon JM, Skene A, Griffith C, Boeddinghaus I, Salter J, Detre S, Hills M (2005). Short-term changes in Ki-67 during neoadjuvant treatment of primary breast cancer with anastrozole or tamoxifen alone or combined correlate with recurrence-free survival. Clinical Cancer Research.

[bib39] Dowsett M, Smith IE, Ebbs SR, Dixon JM, Skene A, A'Hern R, Salter J, Detre S, Hills M, Walsh G (2007). Prognostic value of Ki67 expression after short-term presurgical endocrine therapy for primary breast cancer. Journal of the National Cancer Institute.

[bib40] Dowsett M, Allred C, Knox J, Quinn E, Salter J, Wale C, Cuzick J, Houghton J, Williams N, Mallon E (2008). Relationship between quantitative estrogen and progesterone receptor expression and human epidermal growth factor receptor 2 (HER-2) status with recurrence in the arimidex, tamoxifen, alone or in combination trial. Journal of Clinical Oncology.

[bib41] Dowsett M, Cuzick J, Ingle J, Coates A, Forbes J, Bliss J, Buyse M, Baum M, Buzdar A, Colleoni M (2010). Meta-analysis of breast cancer outcomes in adjuvant trials of aromatase inhibitors versus tamoxifen. Journal of Clinical Oncology.

[bib42] Dowsett M, Nielsen TO, A'Hern R, Bartlett J, Coombes RC, Cuzick J, Ellis M, Henry NL, Hugh JC, Lively T (2011a). Assessment of Ki67 in breast cancer: recommendations from the International Ki67 in Breast Cancer Working Group. Journal of the National Cancer Institute.

[bib43] Dowsett M, Smith I, Robertson J, Robison L, Pinhel I, Johnson L, Salter J, Dunbier A, Anderson H, Ghazoui Z (2011b). Endocrine therapy, new biologicals, and new study designs for presurgical studies in breast cancer. Journal of the National Cancer Institute. Monographs.

[bib44] Dubsky PC, Jakesz R, Mlineritsch B, Postlberger S, Samonigg H, Kwasny W, Tausch C, Stöger H, Haider K, Fitzal F (2012). Tamoxifen and anastrozole as a sequencing strategy: a randomized controlled trial in postmenopausal patients with endocrine-responsive early breast cancer From the Austrian Breast and Colorectal Cancer Study Group. Journal of Clinical Oncology.

[bib45] Eastell R, Adams JE, Coleman RE, Howell A, Hannon RA, Cuzick J, Mackey JR, Beckmann MW, Clack G (2008). Effect of anastrozole on bone mineral density: 5-year results from the anastrozole, tamoxifen, alone or in combination trial 18233230. Journal of Clinical Oncology.

[bib46] Eiermann W, Paepke S, Appfelstaedt J, LlombartCussac A, Eremin J, Vinholes J, Mauriac L, Ellis M, Lassus M, Chaudri-Ross HA (2001). Preoperative treatment of postmenopausal breast cancer patients with letrozole: a randomized double-blind multicenter study. Annals of Oncology.

[bib47] Ellis MJ, Coop A, Singh B, Mauriac L, Llombert-Cussac A, Janicke F, Miller WR, Evans DB, Dugan M, Brady C (2001). Letrozole is more effective neoadjuvant endocrine therapy than tamoxifen for ErbB-1- and/or ErbB-2-positive, estrogen receptor-positive primary breast cancer: evidence from a phase III randomized trial. Journal of Clinical Oncology.

[bib48] Ellis MJ, Gao F, Dehdashti F, Jeffe DB, Marcom PK, Carey LA, Dickler MN, Silverman P, Fleming GF, Kommareddy A (2009). Lower-dose vs high-dose oral estradiol therapy of hormone receptor-positive, aromatase inhibitor-resistant advanced breast cancer a phase 2 randomized study. Journal of the American Medical Association.

[bib49] Engan T, Krane J, Johannessen DC, Lonning PE, Kvinnsland S (1995). Plasma changes in breast cancer patients during endocrine therapy – lipid measurements and nuclear magnetic resonance (NMR) spectroscopy. Breast Cancer Research and Treatment.

[bib50] Ewertz M, Gray KP, Regan MM, Ejlertsen B, Price KN, Thurlimann B, Bonnefoi H, Forbes JF, Paridaens RJ, Rabaglio M (2012). Obesity and risk of recurrence or death after adjuvant endocrine therapy with letrozole or tamoxifen in the Breast International Group 1–98 trial. Journal of Clinical Oncology.

[bib51] Fedele P, Calvani N, Marino A, Orlando L, Schiavone P, Quaranta A, Cinieri S (2012). Targeted agents to reverse resistance to endocrine therapy in metastatic breast cancer: where are we now and where are we going?. Critical Reviews in Oncology/Hematology.

[bib52] Finn RS, Crown JP, Lang I, Boer K, Bondarenko IM, Kulyk SO, Ettl J, Patel R, Pinter T, Schmidt M (2012). Results of a randomized phase 2 study of PD 0332991, a cyclin-dependent kinase (CDK) 4/6 inhibitor, in combination with letrozole vs letrozole alone for first-line treatment of ER+/HER2− advanced breast cancer (BC). Cancer Research.

[bib53] Fisher B, Dignam J, Bryant J, Decillis A, Wickerham DL, Wolmark N, Costantino J, Redmond C, Fisher ER, Bowman DM (1996). Five versus more than five years of tamoxifen therapy for breast cancer patients with negative lymph nodes and estrogen receptor-positive tumors. Journal of the National Cancer Institute.

[bib54] Flageng MH, Moi LL, Dixon JM, Geisler J, Lien EA, Miller WR,  Lønning PE, Mellgren G (2009). Nuclear receptor co-activators and HER-2/neu are upregulated in breast cancer patients during neo-adjuvant treatment with aromatase inhibitors. British Journal of Cancer.

[bib55] Folkerd EJ, Dixon JM, Renshaw L, A'Hern RP, Dowsett M (2012). Suppression of plasma estrogen levels by letrozole and anastrozole is related to body mass index in patients with breast cancer. Journal of Clinical Oncology.

[bib56] Forbes JF, Cuzick J, Buzdar A, Howell A, Tobias JS, Baum M (2008). Effect of anastrozole and tamoxifen as adjuvant treatment for early-stage breast cancer: 100-month analysis of the ATAC trial. Lancet Oncology.

[bib57] Fox EM, Andrade J, Shupnik MA (2009). Novel actions of estrogen to promote proliferation: Integration of cytoplasmic and nuclear pathways. Steroids.

[bib58] Fracchia AA, Randall HT, Farrow JH (1967). The results of adrenalectomy in advanced breast cancer in 500 consecutive patients. Surgery, Gynecology & Obstetrics.

[bib59] Fracchia AA, Farrow JH, Miller TR, Tollefsen RH, Greenberg EJ, Knapper WH (1971). Hypophysectomy as compared with adrenalectomy in the treatment of advanced carcinoma of the breast. Surgery, Gynecology & Obstetrics.

[bib60] Geisler J, Lønning PE (2005). Aromatase inhibition: translation into a successful therapeutic approach. Clinical Cancer Research.

[bib61] Geisler J, Haarstad H, Gundersen S, Raabe N, Kvinnsland S, Lonning PE (1995). Influence of treatment with the anti-oestrogen 3-hydroxytamoxifen (droloxifene) on plasma sex hormone levels in postmenopausal patients with breast cancer. Journal of Endocrinology.

[bib62] Geisler J, Johannessen DC, Anker G, Lønning PE (1996a). Treatment with formestane alone and in combination with aminoglutethimide in heavily pretreated cancer patients: clinical and endocrine effects. European Journal of Cancer.

[bib63] Geisler J, King N, Dowsett M, Ottestad L, Lundgren S, Walton P, Kormeset PO, Lønning PE (1996b). Influence of anastrozole (arimidex), a selective, non-steroidal aromatase inhibitor, on *in vivo* aromatisation and plasma oestrogen levels in postmenopausal women with breast cancer. British Journal of Cancer.

[bib64] Geisler J, King N, Anker G, Ornati G, Di Salle E, Lønning PE, Dowsett M (1998). *In vivo* inhibition of aromatization by exemestane, a novel irreversible aromatase inhibitor, in postmenopausal breast cancer patients. Clinical Cancer Research.

[bib65] Geisler J, Detre S, Berntsen H, Ottestad L, Lindtjørn B, Dowsett M, Lønning P (2001). Influence of neoadjuvant anastrozole (Arimidex) on intratumoral estrogen levels and proliferation markers in patients with locally advanced breast cancer. Clinical Cancer Research.

[bib66] Geisler J, Haynes B, Anker G, Dowsett M, Lønning PE (2002). Influence of letrozole (Femara) and anastrozole (Arimidex) on total body aromatization and plasma estrogen levels in postmenopausal breast cancer patients evaluated in a randomized, cross-over-designed study. Journal of Clinical Oncology.

[bib67] Geisler J, Lønning PE, Krag LE, Løkkevik E, Risberg T, Hagen AI, Schlichting E, Lien EA, Ofjord ES, Eide GE (2006). Changes in bone and lipid metabolism in postmenopausal women with early breast cancer after terminating 2-year treatment with exemestane: a randomised, placebo-controlled study. European Journal of Cancer.

[bib68] Geisler J, Ekse D, Helle H, Duong N, Lønning P (2008a). An optimised, highly sensitive radioimmunoassay for the simultaneous measurement of estrone, estradiol and estrone sulfate in the ultra-low range in human plasma samples. Journal of Steroid Biochemistry and Molecular Biology.

[bib69] Geisler J, Helle H, Ekse D, Duong NK, Evans DB, Nordbø Y, Aas T, Lønning PE (2008b). Letrozole is superior to anastrozole in suppressing breast cancer tissue and plasma estrogen levels. Clinical Cancer Research.

[bib70] Gnant M, Mlineritsch B, Luschin-Ebengreuth G, Kainberger F, Kässmann H, Piswanger-Sölkner JC, Seifert M, Ploner F, Menzel C, Dubsky P (2008). Adjuvant endocrine therapy plus zoledronic acid in premenopausal women with early-stage breast cancer: 5-year follow-up of the ABCSG-12 bone-mineral density substudy. Lancet Oncology.

[bib71] Goss PE, Ingle JN, Martino S, Robert NJ, Muss HB, Piccart MJ, Castiglione M, Tu D, Shepherd LE, Pritchard KI (2005). Randomized trial of letrozole following tamoxifen as extended adjuvant therapy in receptor-positive breast cancer: updated findings from NCICCTG MA.17. Journal of the National Cancer Institute.

[bib72] Harris AL, Cantwell BMJ, Dowsett M (1988). High dose ketoconazole: endocrine and therapeutic effects in postmenopausal breast cancer. British Journal of Cancer.

[bib73] Haynes BP, Straume AH, Geisler J, A'Hern R, Helle H, Smith IE, Lønning PE, Dowsett M (2010). Intratumoral estrogen disposition in breast cancer. Clinical Cancer Research.

[bib74] Howell A, Robertson JF, Quaresma Albano J, Aschermannova A, Mauriac L, Kleeberg UR, Vergote I, Erikstein B, Webster A, Morris C (2002). Fulvestrant, formerly ICI 182,780, is as effective as anastrozole in postmenopausal women with advanced breast cancer progressing after prior endocrine treatment. Journal of Clinical Oncology.

[bib75] Howell A, Robertson JF, Abram P, Lichinitser MR, Elledge R, Bajetta E, Watanabe T, Morris C, Webster A, Dimery I (2004). Comparison of fulvestrant versus tamoxifen for the treatment of advanced breast cancer in postmenopausal women previously untreated with endocrine therapy: a multinational, double-blind, randomized trial. Journal of Clinical Oncology.

[bib76] Huggins C, Dao TL-Y (1953). Adrenalectomy and oophorectomy in treatment of advanced carcinoma of the breast. Journal of the American Medical Association.

[bib77] Hulley S, Grady D, Bush T, Furberg C, Herrington D, Riggs B, Vittinghoff E (1998). Randomized trial of estrogen plus progestin for secondary prevention of coronary heart disease in postmenopausal women. Journal of the American Medical Association.

[bib78] Ingle JN, Schaid DJ, Goss PE, Liu M, Mushiroda T, Chapman JA, Kubo M, Jenkins GD, Batzler A, Shepherd L (2010). Genome-wide associations and functional genomic studies of musculoskeletal adverse events in women receiving aromatase inhibitors. Journal of Clinical Oncology.

[bib79] Jacinto E, Loewith R, Schmidt A, Lin S, Rüegg MA, Hall A, Hall MN (2004). Mammalian TOR complex 2 controls the actin cytoskeleton and is rapamycin insensitive. Nature Cell Biology.

[bib80] Jacobs S, Lønning PE, Haynes B, Griggs L, Dowsett M (1991). Measurement of aromatisation by a urine technique suitable for the evaluation of aromatase inhibitors *in vivo*. Journal of Enzyme Inhibition.

[bib81] Jakesz R, Jonat W, Gnant M, Mittlboeck M, Greil R, Tausch C, Hilfrich J, Kwasny W, Menzel C, Samonigg H (2005). Switching of postmenopausal women with endocrine-responsive early breast cancer to anastrozole after 2 years' adjuvant tamoxifen: combined results of ABCSG trial 8 and ARNO 95 trial. Lancet.

[bib82] Jakesz R, Greil R, Gnant M, Schmid M, Kwasny W, Kubista E, Mlineritsch B, Tausch C, Stierer M, Hofbauer F (2007). Extended adjuvant therapy with anastrozole among postmenopausal breast cancer patients: results from the randomized Austrian Breast and Colorectal Cancer Study Group trial 6a. Journal of the National Cancer Institute.

[bib83] Janes MR, Limon JJ, So L, Chen J, Lim RJ, Chavez MA, Vu C, Lilly MB, Mallya S, Ong ST (2010). Effective and selective targeting of leukemia cells using a TORC1/2 kinase inhibitor. Nature Medicine.

[bib84] Jelovac D, Sabnis G, Long BJ, Macedo L, Goloubeva OG, Brodie AMH (2005). Activation of mitogen-activated protein kinase in xenografts and cells during prolonged treatment with aromatase inhibitor letrozole. Cancer Research.

[bib85] Johannessen DC, Engan T, Di Salle E, Zurlo MG, Paolini J, Ornati G, Piscitelli G, Kvinnsland S, Lonning PE (1997). Endocrine and clinical effects of exemestane (PNU 155971), a novel steroidal aromatase inhibitor, in postmenopausal breast cancer patients: a phase I study. Clinical Cancer Research.

[bib86] Johnell O, Kanis JA, Odén A, Sernbo I, Redlund-Johnell I, Petterson C, De Laet C, Jönsson B (2004). Mortality after osteoporotic fractures. Osteoporosis International.

[bib87] Johnston S, Pippen J, Pivot X, Lichinitser M, Sadeghi S, Dieras V, Gomez HL, Romieu G, Manikhas A, Kennedy MJ (2009). Lapatinib combined with letrozole versus letrozole and placebo as first-line therapy for postmenopausal hormone receptor-positive metastatic breast cancer. Journal of Clinical Oncology.

[bib88] Jones AL, MacNeill F, Jacobs S, Lønning PE, Dowsett M, Powles TJ (1992). The influence of intramuscular 4-hydroxyandrostenedione on peripheral aromatisation in breast cancer patients. European Journal of Cancer.

[bib89] Jones ME, Thorburn AW, Britt KL, Hewitt KN, Misso ML, Wreford NG, Proietto J, Oz OK, Leury BJ, Robertson KM (2001). Aromatase-deficient (ArKO) mice accumulate excess adipose tissue. Journal of Steroid Biochemistry and Molecular Biology.

[bib90] Jordan NJ, Gee JMW, Barrow D, Wakeling AE, Nicholson RI (2004). Increased constitutive activity of PKB/Akt in tamoxifen resistant breast cancer MCF-7 cells. Breast Cancer Research and Treatment.

[bib91] Kanis JA, Johnell O, De Laet C, Jonsson B, Oden A, Ogelsby AK (2002). International variation in hip fracture probabilities: implications for risk assessment. Journal of Bone and Mineral Research.

[bib92] Kaufman B, Mackey JR, Clemens MR, Bapsy PP, Vaid A, Wardley A, Tjulandin S, Jahn M, Lehle M, Feyereislova A (2009). Trastuzumab plus anastrozole versus anastrozole alone for the treatment of postmenopausal women with human epidermal growth factor receptor 2-positive, hormone receptor-positive metastatic breast cancer: results from the Randomized Phase III TAnDEM Study. Journal of Clinical Oncology.

[bib93] Kaufmann M, Jonat W, Hilfrich J, Eidtmann H, Gademann G, Zuna I (2007). Improved overall survival in postmenopausal women with early breast cancer after anastrozole initiated after treatment with tamoxifen compared with continued tamoxifen: the ARNO 95 study. Journal of Clinical Oncology.

[bib94] Kendall A, Dowsett M, Folkerd E, Smith I (2006). Caution: vaginal estradiol appears to be contraindicated in postmenopausal women on adjuvant aromatase inhibitors. Annals of Oncology.

[bib95] Key TJ, Appleby PN, Reeves GK, Roddam A, Dorgan JF, Longcope C, Stanczyk FZ, Stephenson HE, Falk RT, Miller R (2003). Body mass index, serum sex hormones, and breast cancer risk in postmenopausal women. Journal of the National Cancer Institute.

[bib96] Knowlden JM, Hutcheson IR, Jones HE, Madden T, Gee JM, Harper ME, Barrow D, Wakeling AE, Nicholson RI (2003). Elevated levels of epidermal growth factor receptor/c-erbB2 heterodimers mediate an autocrine growth regulatory pathway in tamoxifen-resistant MCF-7 cells. Endocrinology.

[bib97] Kofman S, Nagamani D, Buenger RF, Taylor SG (1958). The use of prednisolone in the treatment of disseminated breast carcinoma. Cancer.

[bib98] Kwan ML, Chen WY, Kroenke CH, Weltzien EK, Beasley JM, Nechuta SJ, Poole EM, Lu W, Holmes MD, Quesenberry CP (2012). Pre-diagnosis body mass index and survival after breast cancer in the After Breast Cancer Pooling Project. Breast Cancer Research and Treatment.

[bib99] van Landeghem AJJ, Poortman J, Nabuurs M, Thijssen JHH (1985). Endogenous concentration and subcellular distribution of estrogens in normal and malignant breast tissue. Cancer Research.

[bib100] Lemon HM (1959). Prednisone therapy of advanced mammary cancer. Cancer.

[bib101] Luft R, Olivecrona H, Sjögren B (1952). Hypophysectomy in man. Nordisk Medicin.

[bib102] Lønning PE (2003). Study of suboptimum treatment response: lessons from breast cancer. Lancet Oncology.

[bib103] Lønning PE (2004). Aromatase inhibitors in breast cancer. Endocrine-Related Cancer.

[bib104] Lønning PE (2006). Comparing cost/utility of giving an aromatase inhibitor as monotherapy for 5 years versus sequential administration following 2–3 or 5 years of tamoxifen as adjuvant treatment for postmenopausal breast cancer. Annals of Oncology.

[bib105] Lønning PE, Ekse D (1995). A sensitive assay for measurement of plasma estrone sulphate in patients on treatment with aromatase inhibitors. Journal of Steroid Biochemistry and Molecular Biology.

[bib106] Lønning PE, Geisler J (2008). Indications and limitations of third-gene ration aromatase inhibitors. Expert Opinion on Investigational Drugs.

[bib107] Lønning PE, Knappskog S (2013). Mapping genetic alterations causing chemoresistance in cancer; identifying the roads by tracking the drivers. Oncogene.

[bib108] Lønning PE, Kvinnsland S (1988). Mechanisms of action of aminoglutethimide as endocrine therapy of breast cancer. Drugs.

[bib109] Lønning PE, Kvinnsland S, Thorsen T, Ueland PM (1987). Alterations in the metabolism of oestrogens during treatment with aminoglutethimide in breast cancer patients. Preliminary findings. Clinical Pharmacokinetics.

[bib110] Lønning PE, Johannessen DC, Thorsen T (1989a). Alterations in the production rate and the metabolism of oestrone and oestrone sulphate in breast cancer patients treated with aminoglutethimide. British Journal of Cancer.

[bib185] Lønning PE, Jacobs S, Jones A, Haynes B, Powles T, Dowsett M (1991). The influence of CGS 16949A on peripheral aromatisation in breast cancer patients. British Journal of Cancer.

[bib111] Lønning PE, Skulstad P, Sunde A, Thorsen T (1989b). Separation of urinary metabolites of radiolabelled estrogens in man by HPLC. Journal of Steroid Biochemistry.

[bib112] Lønning PE, Dowsett M, Powles TJ (1990). Postmenopausal estrogen synthesis and metabolism: alterations caused by aromatase inhibitors used for the treatment of breast cancer. Journal of Steroid Biochemistry.

[bib113] Lønning PE, Bajetta E, Murray R, Tubiana-Hulin M, Eisenberg PD, Mickiewicz E, Celio L, Pitt P, Mita M, Aaronson NK (2000). Activity of exemestane in metastatic breast cancer after failure of nonsteroidal aromatase inhibitors: a phase II trial. Journal of Clinical Oncology.

[bib114] Lønning PE, Taylor PD, Anker G, Iddon J, Wie L, Jørgensen LM, Mella O, Howell A (2001). High-dose estrogen treatment in postmenopausal breast cancer patients heavily exposed to endocrine therapy. Breast Cancer Research and Treatment.

[bib115] Lønning PE, Geisler J, Krag LE, Erikstein B, Bremnes Y, Hagen AI, Schlichting E, Lien EA, Ofjord ES, Paolini J (2005). Effects of exemestane administered for 2 years versus placebo on bone mineral density, bone biomarkers, and plasma lipids in patients with surgically resected early breast cancer. Journal of Clinical Oncology.

[bib116] Lønning PE, Helle H, Duong NK, Ekse D, Aas T, Geisler J (2009). Tissue estradiol is selectively elevated in receptor positive breast cancers while tumour estrone is reduced independent of receptor status. Journal of Steroid Biochemistry and Molecular Biology.

[bib117] Lønning PE, Haynes BP, Straume AH, Dunbier A, Helle H, Knappskog S, Dowsett M (2011). Exploring breast cancer estrogen disposition: the basis for endocrine manipulation. Clinical Cancer Research.

[bib118] MacNeill FA, Jones AL, Jacobs S, Lønning PE, Powles TJ, Dowsett M (1992). The influence of aminoglutethimide and its analogue rogletimide on peripheral aromatisation in breast cancer. British Journal of Cancer.

[bib119] MacNeill FA, Jacobs S, Lønning PE, Powles TJ, Dowsett M (1994). Combined treatment with 4-hydroxyandrostenedione and aminoglutethimide: effects on aromatase inhibition and oestrogen suppression. British Journal of Cancer.

[bib120] MacNeill FA, Jacobs S, Dowsett M, Lonning PE, Powles TJ (1995). The effects of oral 4-hydroxyandrostenedione on peripheral aromatisation in post-menopausal breast cancer patients. Cancer Chemotherapy and Pharmacology.

[bib121] Mamounas EP, Jeong JH, Wickerham DL, Smith RE, Ganz PA, Land SR, Eisen A, Fehrenbacher L, Farrar WB, Atkins JN (2008). Benefit from exemestane as extended adjuvant therapy after 5 years of adjuvant tamoxifen: intention-to-treat analysis of the National Surgical Adjuvant Breast And Bowel Project B-33 trial. Journal of Clinical Oncology.

[bib122] Manson JE, Hsia J, Johnson KC, Rossouw JE, Assaf AR, Lasser NL, Trevisan M, Black HR, Heckbert SR, Detrano R (2003). Estrogen plus progestin and the risk of coronary heart disease. New England Journal of Medicine.

[bib123] Masamura S, Santner SJ, Heitjan DF, Santen RJ (1995). Estrogen deprivation causes estradiol hypersensitivity in human breast cancer cells. Journal of Clinical Endocrinology and Metabolism.

[bib124] Meldrum DR, Davidson BJ, Tataryn IV, Judd HL (1981). Changes in circulating steroids with aging in postmenopausal women. Obstetrics and Gynecology.

[bib125] Mendelson CR, Jiang B, Shelton JM, Richardson JA, Hinshelwood MM (2005). Transcriptional regulation of aromatase in placenta and ovary. Journal of Steroid Biochemistry and Molecular Biology.

[bib126] Mieog JSD, Morden JP, Bliss JM, Coombes RC, van de Velde CJH, Comm IESS (2012). Carpal tunnel syndrome and musculoskeletal symptoms in postmenopausal women with early breast cancer treated with exemestane or tamoxifen after 2–3 years of tamoxifen: a retrospective analysis of the Intergroup Exemestane Study. Lancet Oncology.

[bib127] Miller WR, Telford J, Love C, Leonard RCF, Hillier S, Gundacker H, Smith H, Dixon JM (1998). Effects of letrozole as primary medical therapy on in situ oestrogen synthesis and endogenous oestrogen levels within the breast. Breast.

[bib128] Miller WR, Larionov A, Renshaw L, Anderson TJ, Walker JR, Krause A, Sing T, Evans DB, Dixon JM (2009). Gene expression profiles differentiating between breast cancers clinically responsive or resistant to letrozole. Journal of Clinical Oncology.

[bib129] Miller TW, Balko JM, Arteaga CL (2011). Phosphatidylinositol 3-kinase and antiestrogen resistance in breast cancer. Journal of Clinical Oncology.

[bib130] Morales L, Pans S, Paridaens R, Westhovens R, Timmerman D, Verhaeghe J, Wildiers H, Leunen K, Amant F, Berteloot P (2007). Debilitating musculoskeletal pain and stiffness with letrozole and exemestane: associated tenosynovial changes on magnetic resonance imaging. Breast Cancer Research and Treatment.

[bib131] Morales L, Pans S, Verschueren K, Van Calster B, Paridaens R, Westhovens R, Timmerman D, De Smet L, Vergote I, Christiaens MR (2008). Prospective study to assess short-term intra-articular and tenosynovial changes in the aromatase inhibitor-associated arthralgia syndrome. Journal of Clinical Oncology.

[bib132] Morimoto LM, White E, Chen Z, Chlebowski RT, Hays J, Kuller L, Lopez AM, Manson J, Margolis KL, Muti PC (2002). Obesity, body size, and risk of postmenopausal breast cancer: the Women's Health Initiative (United States). Cancer Causes & Control.

[bib133] Morishima A, Grumbach MM, Simpson ER, Fisher C, Qin K (1995). Aromatase deficiency in male and female siblings caused by a novel mutation and the physiological role of estrogens. Journal of Clinical Endocrinology and Metabolism.

[bib134] Motzer RJ, Escudier B, Oudard S, Hutson TE, Porta C, Bracarda S, Grünwald V, Thompson JA, Figlin RA, Hollaender N (2010). Phase 3 trial of everolimus for metastatic renal cell carcinoma: final results and analysis of prognostic factors. Cancer.

[bib135] Mouridsen H, Gershanovich M, Sun Y, Pérez-Carrión R, Boni C, Monnier A, Apffelstaedt J, Smith R, Sleeboom HP, Jänicke F (2001). Superior efficacy of letrozole versus tamoxifen as first-line therapy for postmenopausal women with advanced breast cancer: results of a phase III study of the International Letrozole Breast Cancer Group. Journal of Clinical Oncology.

[bib136] Mouridsen H, Gershanovich M, Sun Y, Perez-Carrion R, Boni C, Monnier A, Apffelstaedt J, Smith R, Sleeboom HP, Jaenicke F (2003). Phase III study of letrozole versus tamoxifen as first-line therapy of advanced breast cancer in postmenopausal women: analysis of survival and update of efficacy from the International Letrozole Breast Cancer Group. Journal of Clinical Oncology.

[bib137] Mouridsen H, Giobbie-Hurder A, Goldhirsch A, Thürlimann B, Paridaens R, Smith I, Mauriac L, Forbes J, Price KN, Regan MM (2009). Letrozole therapy alone or in sequence with tamoxifen in women with breast cancer. New England Journal of Medicine.

[bib138] Niraula S, Ocana A, Ennis M, Goodwin PJ (2012). Body size and breast cancer prognosis in relation to hormone receptor and menopausal status: a meta-analysis. Breast Cancer Research and Treatment.

[bib139] Nishihori T, Choi J, DiGiovanna MP, Thomson JG, Kohler PC, McGurn J, Chung GG (2008). Carpal tunnel syndrome associated with the use of aromatase inhibitors in breast cancer. Clinical Breast Cancer.

[bib140] Osborne CK, Pippen J, Jones SE, Parker LM, Ellis M, Come S, Gertler SZ, May JT, Burton G, Dimery I (2002). Double-blind, randomized trial comparing the efficacy and tolerability of fulvestrant versus anastrozole in postmenopausal women with advanced breast cancer progressing on prior endocrine therapy: results of a North American trial. Journal of Clinical Oncology.

[bib141] Partridge AH, LaFountain A, Mayer E, Taylor BS, Winer E, Asnis-Alibozek A (2008). Adherence to initial adjuvant anastrozole therapy among women with early-stage breast cancer. Journal of Clinical Oncology.

[bib142] Penault-Llorca F, André F, Sagan C, Lacroix-Triki M, Denoux Y, Verriele V, Jacquemier J, Baranzelli MC, Bibeau F, Antoine M (2009). Ki67 expression and docetaxel efficacy in patients with estrogen receptor-positive breast cancer. Journal of Clinical Oncology.

[bib143] Perez EA, Josse RG, Pritchard KI, Ingle JN, Martino S, Findlay BP, Shenkier TN, Tozer RG, Palmer MJ, Shepherd LE (2006). Effect of letrozole versus placebo on bone mineral density in women with primary breast cancer completing 5 or more years of adjuvant tamoxifen: a companion study to NCICCTG MA.17. Journal of Clinical Oncology.

[bib144] Pfeiler G, Königsberg R, Fesl C, Mlineritsch B, Stoeger H, Singer CF, Pöstlberger S, Steger GG, Seifert M, Dubsky P (2011). Impact of body mass index on the efficacy of endocrine therapy in premenopausal patients with breast cancer: an analysis of the prospective ABCSG-12 trial. Journal of Clinical Oncology.

[bib145] Poortman J, Thijssen JHH, Waard FD (1981). Plasma oestrone, oestradiol and androstenedione levels in post-menopausal women: relation to body weight and height. Maturitas.

[bib146] Powles TJ, Hickish T, Kanis JA, Tidy A, Ashley S (1996). Effect of tamoxifen on bone mineral density measured by dual-energy X-ray absorptiometry in healthy premenopausal and postmenopausal women. Journal of Clinical Oncology.

[bib147] Protani M, Coory M, Martin JH (2010). Effect of obesity on survival of women with breast cancer: systematic review and meta-analysis. Breast Cancer Research and Treatment.

[bib148] Rasmussen BB, Regan MM, Lykkesfeldt AE, Dell'Orto P, Del Curto B, Henriksen KL, Mastropasqua MG, Price KN, Méry E, Lacroix-Triki M (2008). Adjuvant letrozole versus tamoxifen according to centrally-assessed ERBB2 status for postmenopausal women with endocrine-responsive early breast cancer: supplementary results from the BIG 1–98 randomised trial. Lancet Oncology.

[bib149] Regan MM, Neven P, Giobbie-Hurder A, Goldhirsch A, Ejlertsen B, Mauriac L, Forbes JF, Smith I, Láng I, Wardley A (2011). Assessment of letrozole and tamoxifen alone and in sequence for postmenopausal women with steroid hormone receptor-positive breast cancer: the BIG 1–98 randomised clinical trial at 8.1 years median follow-up. Lancet Oncology.

[bib150] Robertson JF, Lindemann JP, Llombart-Cussac A, Rolski J, Feltl D, Dewar J, Emerson L, Dean A, Ellis MJ (2012). Fulvestrant 500 mg versus anastrozole 1 mg for the first-line treatment of advanced breast cancer: follow-up analysis from the randomized ‘FIRST’ study. Breast Cancer Research and Treatment.

[bib151] Rochira V, Carani C (2009). Aromatase deficiency in men: a clinical perspective. Nature Reviews. Endocrinology.

[bib152] Sabnis G, Brodie A (2011). Adaptive changes results in activation of alternate signaling pathways and resistance to aromatase inhibitor resistance. Molecular and Cellular Endocrinology.

[bib153] Samojlik E, Veldhuis JD, Wells SA, Santen RJ (1980). Preservation of androgen secretion during estrogen suppression with aminoglutethimide in the treatment of metastatic breast carcinoma. Journal of Clinical Investigation.

[bib154] Santen RJ (1981). Suppression of estrogens with aminoglutethimide and hydrocortisone (medical adrenalectomy) as treatment of advanced breast carcinoma: a review. Breast Cancer Research.

[bib155] Santen RJ, Lipton A, Kendall J (1974). Successful medical adrenalectomy with aminoglutethimide. Journal of the American Medical Association.

[bib156] Santen RJ, Santner S, Davis B, Veldhuis J, Samojlik E, Ruby E (1978). Aminoglutethimide inhibits extraglandular estrogen production in postmenopausal women with breast carcinoma. Journal of Clinical Endocrinology and Metabolism.

[bib157] Santen RJ, Worgul TJ, Samojlik E, Interrante A, Boucher AE, Lipton A, Harvey HA, White DS, Smart E, Cox C (1981). A randomized trial comparing surgical adrenalectomy with aminoglutethimide plus hydrocortisone in women with advanced breast cancer. New England Journal of Medicine.

[bib158] Santen RJ, Song RX, Zhang Z, Kumar R, Jeng MH, Masamura A, Lawrence J, Berstein L, Yue W (2005). Long-term estradiol deprivation in breast cancer cells up-regulates growth factor signaling and enhances estrogen sensitivity. Endocrine-Related Cancer.

[bib159] Sasano H, Anderson TJ, Silverberg SG, Santen RJ, Conway M, Edwards DP, Krause A, Bhatnagar AS, Evans DB, Miller WR (2005). The validation of new aromatase monoclonal antibodies for immunohistochemistry – a correlation with biochemical activities in 46 cases of breast cancer. Journal of Steroid Biochemistry and Molecular Biology.

[bib160] Semiglazov V, Kletsel A, Semiglazov V, Zhiltzova E, Ivanov V, Dashyan G, Bozhok A, Melnikova O, Paltuev R, Berstein L (2005). Exemestane (E) versus tamoxifen (T) as neoadjuvant endocrine therapy for postmenopausal women with ER+ breast cancer (T2N1–2, T3N0–1, T4N0M0). Journal of Clinical Oncology.

[bib161] Serra V, Scaltriti M, Prudkin L, Eichhorn PJ, Ibrahim YH, Chandarlapaty S, Markman B, Rodriguez O, Guzman M, Rodriguez S (2011). PI3K inhibition results in enhanced HER signaling and acquired ERK dependency in HER2-overexpressing breast cancer. Oncogene.

[bib162] Sestak I, Sapunar F, Cuzick J (2009). Aromatase inhibitor-induced carpal tunnel syndrome: results from the ATAC trial. Journal of Clinical Oncology.

[bib163] Sestak I, Distler W, Forbes JF, Dowsett M, Howell A, Cuzick J (2010). Effect of body mass index on recurrences in tamoxifen and anastrozole treated women: an exploratory analysis from the ATAC trial. Journal of Clinical Oncology.

[bib164] Sluijmer AV, Heineman MA, Jong FHD, Evers JLH (1995). Endocrine activity of the postmenopausal ovary: the effects of pituitary down-regulation and oophorectomy. Journal of Clinical Endocrinology and Metabolism.

[bib165] Smith IE, Dowsett M, Ebbs SR, Dixon JM, Skene A, Blohmer JU, Ashley SE, Francis S, Boeddinghaus I, Walsh G (2005). Neoadjuvant treatment of postmenopausal breast cancer with anastrozole, tamoxifen, or both in combination: the Immediate Preoperative Anastrozole, Tamoxifen, or Combined with Tamoxifen (IMPACT) multicenter double-blind randomized trial. Journal of Clinical Oncology.

[bib166] Sørlie T, Perou CM, Tibshirani R, Aas T, Geisler S, Johnsen H, Hastie T, Eisen MB, van de Rijn M, Jeffrey SS (2001). Gene expression patterns of breast carcinomas distinguish tumor subclasses with clinical implications. PNAS.

[bib167] Stewart HJ, Prescott RJ, Forrest APM (2001). Scottish adjuvant tamoxifen trial: a randomized study updated to 15 years. Journal of the National Cancer Institute.

[bib168] Tormey DC, Gray R, Falkson HC (1996). Postchemotherapy adjuvant tamoxifen therapy beyond five years in patients with lymph node-positive breast cancer. Journal of the National Cancer Institute.

[bib169] Trial TW (1995). Effects of estrogen or estrogen/progestin regimens on heart disease risk factors in postmenopausal women. The postmenopausal estrogen/progestin interventions (PEPI) trial. Journal of the American Medical Association.

[bib170] Untch M, Gelber RD, Jackisch C, Procter M, Baselga J, Bell R, Cameron D, Bari M, Smith I, Leyland-Jones B (2008). Estimating the magnitude of trastuzumab effects within patient subgroups in the HERA trial. Annals of Oncology.

[bib171] Vallabhaneni S, Nair BC, Cortez V, Challa R, Chakravarty D, Tekmal RR, Vadlamudi RK (2011). Significance of ER–Src axis in hormonal therapy resistance. Breast Cancer Research and Treatment.

[bib172] van de Velde CJ, Rea D, Seynaeve C, Putter H, Hasenburg A, Vannetzel JM, Paridaens R, Markopoulos C, Hozumi Y, Hille ET (2011). Adjuvant tamoxifen and exemestane in early breast cancer (TEAM): a randomised phase 3 trial. Lancet.

[bib173] Villarreal-Garza C, Cortes J, Andre F, Verma S (2012). mTOR inhibitors in the management of hormone receptor-positive breast cancer: the latest evidence and future directions. Annals of Oncology.

[bib174] Wasan KM, Goss PE, Pritchard PH, Shepherd L, Palmer MJ, Liu S, Tu D, Ingle JN, Heath M, Deangelis D (2005). The influence of letrozole on serum lipid concentrations in postmenopausal women with primary breast cancer who have completed 5 years of adjuvant tamoxifen (NCIC CTG MA.17L). Annals of Oncology.

[bib175] Weigel MT, Banerjee S, Arnedos M, Salter J, A'Hern R, Dowsett M, Martin LA (2013). Enhanced expression of the PDGFR/Abl signaling pathway in aromatase inhibitor-resistant breast cancer. Annals of Oncology.

[bib176] Weigelt B, Warne PH, Downward J (2011). PIK3CA mutation, but not PTEN loss of function, determines the sensitivity of breast cancer cells to mTOR inhibitory drugs. Oncogene.

[bib177] Wills S, Ravipati A, Venuturumilli P, Kresge C, Folkerd E, Dowsett M, Hayes DF, Decker DA (2012). Effects of vaginal estrogens on serum estradiol levels in postmenopausal breast cancer survivors and women at risk of breast cancer taking an aromatase inhibitor or a selective estrogen receptor modulator. Journal of Oncology Practice.

[bib178] Wolff AC, Lazar AA, Bondarenko I, Garin AM, Brincat S, Chow L, Sun Y, Neskovic-Konstantinovic Z, Guimaraes RC, Fumoleau P (2013). Randomized phase III placebo-controlled trial of letrozole plus oral temsirolimus as first-line endocrine therapy in postmenopausal women with locally advanced or metastatic breast cancer. Journal of Clinical Oncology.

[bib179] Yao JC, Lombard-Bohas C, Baudin E, Kvols LK, Rougier P, Ruszniewski P, Hoosen S, St Peter J, Haas T, Lebwohl D (2010). Daily oral everolimus activity in patients with metastatic pancreatic neuroendocrine tumors after failure of cytotoxic chemotherapy: a phase II trial. Journal of Clinical Oncology.

[bib180] Zhou J, Suzuki T, Kovacic A, Saito R, Miki Y, Ishida T, Moriya T, Simpson ER, Sasano H, Clyne CD (2005). Interactions between prostaglandin E-2, liver receptor homologue-1, and aromatase in breast cancer. Cancer Research.

